# Top Notch Targeting Strategies in Cancer: A Detailed Overview of Recent Insights and Current Perspectives

**DOI:** 10.3390/cells9061503

**Published:** 2020-06-20

**Authors:** Gillian Moore, Stephanie Annett, Lana McClements, Tracy Robson

**Affiliations:** 1 School of Pharmacy and Biomolecular Sciences, Irish Centre for Vascular Biology, Royal College of Surgeons, D02 YN77 Dublin, Ireland; gillianmoore@rcsi.com (G.M.); stephanieannett@rcsi.com (S.A.); 2 The School of Life Sciences, Faculty of Science, University of Technology Sydney, Sydney, NSW 2007, Australia; lana.mcclements@uts.edu.au

**Keywords:** notch antibodies, gamma secretase inhibitors, cancer stem cells, drug resistance, clinical trial, breast tumours, lung tumours, leukaemias, desmoid tumours, adenoid cystic carcinoma

## Abstract

Evolutionarily conserved Notch plays a critical role in embryonic development and cellular self-renewal. It has both tumour suppressor and oncogenic activity, the latter of which is widely described. Notch-activating mutations are associated with haematological malignancies and several solid tumours including breast, lung and adenoid cystic carcinoma. Moreover, upregulation of Notch receptors and ligands and aberrant Notch signalling is frequently observed in cancer. It is involved in cancer hallmarks including proliferation, survival, migration, angiogenesis, cancer stem cell renewal, metastasis and drug resistance. It is a key component of cell-to-cell interactions between cancer cells and cells of the tumour microenvironment, such as endothelial cells, immune cells and fibroblasts. Notch displays diverse crosstalk with many other oncogenic signalling pathways, and may drive acquired resistance to targeted therapies as well as resistance to standard chemo/radiation therapy. The past 10 years have seen the emergence of different classes of drugs therapeutically targeting Notch including receptor/ligand antibodies, gamma secretase inhibitors (GSI) and most recently, the development of Notch transcription complex inhibitors. It is an exciting time for Notch research with over 70 cancer clinical trials registered and the first-ever Phase III trial of a Notch GSI, nirogacestat, currently at the recruitment stage.

## 1. Introduction: Notch-Signalling Pathway and Downstream Transcription Targets

Throughout evolution, establishment and maintenance of cell-to-cell interactions via signalling transduction, pathways have been critical for the development of multicellular organisms. Large numbers of studies conducted in Drosophila, Caenorhabditis, vertebrates and more recently putative primitive Metazoans, such as sponges [1,2], have indicated that the Notch pathway is widely conserved and is an important, if not the most important orchestrator of higher-level cellular interactions and organisation associated with the emergence of multicellular organisms. In vertebrates, Notch plays a fundamental role in embryonic development [2] and thereafter in cellular self-renewal processes and homeostasis throughout life, controlling proliferation, differentiation, cell fate, apoptosis or cell death. While the core Notch-signalling pathway looks relatively simple, owing to the small number of specific proteins involved, it is, in fact, a very sophisticated and adaptable system due to the widespread cellular responses it can trigger.

**Figure 1:**
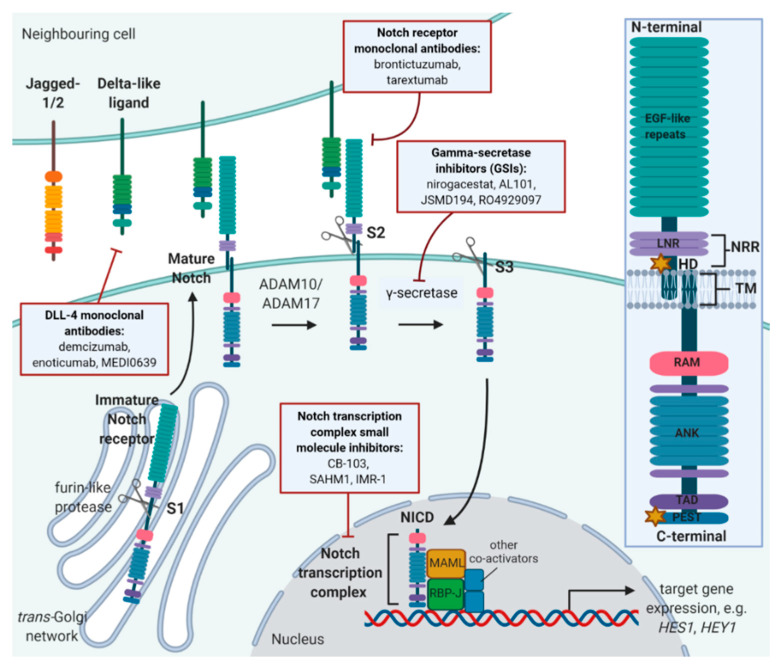
Notch signalling and strategies for pharmacological targeting of this pathway. Right Inset: Notch receptors consist of N-terminal EGF-like repeats, followed by three Lin12/Notch repeats (LNR) and the heterodimerisation (HD) domain, which together form the negative regulatory region (NRR). Next, there is a single transmembrane (TM) repeat, followed by an intracellular RBP-J-associated molecule (RAM), ankyrin repeats (ANK), a transcriptional TAD domain and a degradation PEST domain. Notch-activating mutations (labelled with the star) are commonly found in the HD and PEST domain. Main diagram: Immature Notch receptor is processed in the *trans-*Golgi network where a furin-like protease cleaves it at Site S1 within the HD domain, located between the LNR and the TM, creating the mature heterodimeric Notch receptor, sustained by NRR. Notch-specific ligands located on a neighbouring cell (e.g., Jagged-1/2 or Delta-like ligand (DLL-) 1/3/4) interact with the N-terminal of the mature Notch receptor, causing activation (i.e., release of its autoinhibited conformation). ADAM 10/17 matrix metalloproteinases cleave Notch at Site S2 located in TM (extracellular side). The remaining TM domain is cleaved by a gamma secretase at Site S3 (intracellular side). Notch intracellular domain (NICD) is released and translocates to the nucleus where it forms a complex with DNA-binding transcription factors RBP-J (CSL), mastermind-like (MAML) protein and other coactivator proteins and enzymes, and stimulates transcription of Notch target genes including the hairy/enhance of split 1 (HES) and Hes-related (HEY) families of transcription repressors, such as *HES1* and *HEY1*. There are numerous strategies to pharmacologically target Notch signalling including Notch receptor monoclonal antibodies, ligand-targeted antibodies (e.g., DLL-4 antibodies), gamma secretase inhibitors (GSIs) and Notch transcription complex small-molecule inhibitors.

The mammalian Notch pathway consists of four single-pass transmembrane protein receptors: Notches 1, 2, 3 and 4. All Notch receptors have a common extracellular N-terminal epidermal growth factor (EGF)-repeat. This is followed by three LNR (Lin12/Notch) repeats and a juxtamembrane heterodimerisation (HD) domain, which together constitute a negative regulatory region (NRR) [3,4]. Next, there is a single transmembrane (TM) repeat, followed by the intracellular C-terminal domain consisting of RAM (RBP-J-associated molecule) and ANK (ankyrin repeats), in addition to TAD and PEST domains required for transcription and degradation activities, respectively (see references for in-depth reviews of receptor structure: [5,6]; Figure 1). While Notch 1 and Notch 2 are ubiquitously expressed throughout development and in adult life, Notch 3 and Notch 4 are the most abundant in cell subtypes of the vasculature, such as smooth muscle cells, pericytes and endothelial cells. Notch 1 and Notch 2 knockouts are embryonically lethal with multiple organ defects observed, while Notch 3 and Notch 4 knockouts are both viable but display subtle vascular abnormalities [7,8,9]. There are five ligands involved in the activation of mammalian Notch receptors: Delta-like ligand (DLL-) 1, 3 and 4 and Jagged-(JAG) 1 and 2 [10]. Detailed structural and functional differences between the Notch receptors and ligands has been widely reviewed elsewhere in the literature (refer to [6,10,11,12,13]). During *trans*-Golgi processing and trafficking to the plasma membrane, a newly synthesised Notch receptor undergoes cleavage by a furin-like protease within the HD domain at Site S1 (located between LNR and TM) [4], creating a mature heterodimeric receptor consisting of a noncovalently associated extracellular and transmembrane subunit, sustained by the NRR. The receptor is held in this resting protease-resistant conformation by NRR until such a time that ligand binding occurs [14]. The binding of mature Notch receptor to ligand expressed on the surface of a neighbouring cell, releases that autoinhibited conformation of the NRR, allowing two successive cleavage events to occur and finally yield functional signalling protein. Firstly, ADAM matrix metalloproteinases (ADAM 17 and 10) cleave the TM domain at Site S2, located at the external side of the plasma membrane [15]. Secondly, what remains of the TM is cleaved by a gamma secretase at Site S3, releasing the Notch Intracellular Domain (NICD), which translocates to the nucleus [16]. Here, the NICD forms a complex with the DNA-binding transcription factor RBP-J (also known as CBF-1/Suppressor of Hairless/LAG-1 (CSL)), and the mastermind-like (MAML) proteins, which require an interaction with the RAM and ANK domains of NICD. The complex recruits other transcription coactivators and DNA modification enzymes to the site, ultimately leading to the transcriptional activation of Notch-dependent target genes [17,18,19].

Transcriptional targets include the hairy/enhancer of split (HES) and HES-related (HEY) families of transcription repressors, such as HES-1 and HESR-1 (HES-related repressor protein-1)/HEY-1 [19,20], transcription factors c-Myc [21], NFκB [22] and GATA binding factor 3 (*GATA3*) [23], cell cycle regulators p21 (*CDKN1A*) [24], Cyclin-D1 (*CCND1*) [25], Cyclin-D3 (*CCND3*) [26] and apoptosis regulator Bcl-2 [27]. Aside from canonical Notch signalling detailed above, there is evidence to suggest that NICD interacts or forms a complex with effector proteins other than RBP-J. This is called noncanonical Notch signalling and it can be either ligand-dependent or independent. Notch has been shown to antagonise WNT/β-catenin signalling pathway in progenitor and stem cells, through a post-translational suppression of active β-catenin, independent of its association with RBP-J [28]. Suppression of transforming growth factor-beta (TGF-β) via pharmacological inhibitors has been shown to downregulate Notch activation, and similarly, Notch inhibition downregulates TGF-β signalling [29]. Crosstalk between these two parallel signalling pathways has been shown to occur via a physical interaction between the NICD and the TGF-β downstream signalling mediators called Smads [30]. Other reported noncanonical roles of Notch include regulation of hypoxia-induced signalling pathways via an association with hypoxia-inducible factor 1 alpha (HIF1-α) [31] and pAkt-driven cell survival via an interaction with mTOR-Rictor [32]. Similarly, NFκB activation/signalling, IL-6/STAT signalling, and transcription of oestrogen-receptor-dependent genes require an interaction of Notch with the NFκB regulatory protein complex, IK-β kinase (IKK) [33,34,35,36]. To date, the majority of the literature has described the role of canonical Notch signalling in cancer and the relevance of alternative RBP-J-independent interactions have yet to be fully defined in the context of cancer.

## 2. Notch in Cancer: An Overview

### 2.1. Tumour Suppressor Role in Squamous Cell Carcinomas (SCC)

While early research focused on the role of Notch in developmental processes and homeostasis, in the last 15–20 years emphasis has shifted to understanding the critical role of this pathway in cancer. Notch has been described as both a tumour promoter (oncogene) and a tumour suppressor in the literature, and its role is very much dependent on the cell and tissue context, indicating the complexity of this pathway. The best-studied example of its tumour-suppressive role is in squamous cell carcinomas (SCC) of various tissue types including cutaneous SCC (cSCC) [37,38], bladder SCC [39], lung SCC [38] and oesophageal SCC [40], where the loss-of-function somatic mutations in Notch drive cancer progression. The use of next-generation sequencing has been pivotal in revealing that Notch mutations are among some of the most frequently occurring mutations in SCCs. For example, Wang et al. reported that missense *NOTCH1* mutations (leading to loss-of-function) occur in approximately three-quarters of cSCC cases [38]. These recurrent sequencing patterns in clinical cSCC samples suggest a tumour suppressor role for Notch, which have been verified in numerous in vitro and in vivo studies. For example, in a commonly used chemical carcinogen DMBA-TPA-induced model of cSCC, mice acquire loss-of-function mutations in *Notch1*, indicating an association with cSCC tumour development [41]. Additionally, in an adult mouse model of epidermal keratinocyte *Notch1* deletion, epidermal and corneal hyperplasia was observed followed by the development of skin tumours [42]. The role of Notch in some types of SCC, such as head and neck SCC (HNSCC) has been controversial. One study has reported the detection of *NOTCH1* inactivating mutations in 15% of HNSCC cases, suggesting a tumour suppressor role [43]. However, another study has provided evidence of a bimodal pattern of the Notch pathway in HNSCC, where a small subset of patients harbour Notch inactivating mutations (10–15%) but interestingly, a larger subset (32%) have Notch 1 pathway overexpression and downstream pathway activation [44]. Indeed, a meta-analysis of nine studies, albeit relatively small, indicated overexpression of the Notch pathway in HNSCC, with Notch 1 showing an association with poor differentiation, disease progression and lymph node metastasis [45]. Notch 1 was also predictive of poor overall survival (OS). In some tumour contexts, such as cSCC, there is a rationale for therapeutic restoration and activation of Notch signalling. However, one obvious limitation of this approach is the potential undesired activation of Notch signalling in other tissues where it can be tumourigenic, as is the case for some solid and haematological malignancies.

### 2.2. Oncogenic Notch in Haematological Malignancies: Driver Mutations and Biomarker Potential

The Notch-signalling pathway is involved in several hallmarks of cancer including enhanced proliferation, survival, migration, angiogenesis, metastasis and drug resistance [46]. There is a wide range of Notch-activating mutations and alterations reported in the literature including missense and nonsense mutations, small frame-shifting indels, deletions and translocations, which either interrupt negative regulatory regions in the extracellular portion of the receptor, predominantly the HD domain, or in the intracellular PEST domain [47,48]. Gene translocations or rearrangements that remove a large portion of the extracellular domain or mutations in the HD domain of Notch 1 lead to a dysfunctional NRR with an impaired ability to perform its critical autoinhibitory role, and ultimately ligand-independent proteolytic cleavage and activation of Notch 1 signalling ensues [49]. As mentioned previously, the PEST domain plays an important regulatory role in degrading NICD, preventing excessive Notch activation. However, inactivating mutations in the C-terminal PEST domain of Notch 1 prevents this regulatory role, increasing the half-life of NICD and its window for transcriptional activity. Surprisingly, while most mutations reported are defined as inactivating mutations, these are confined to negative regulatory regions of the receptor, thus leading to an overall gain-in-function effect on Notch receptor signalling. To date, the majority of reported Notch receptor genetic alterations are in Notch 1. The first reported Notch alteration in cancer was a chromosomal translocation of the 3′ region of Notch 1 into the T cell receptor β (TCR-β) locus resulting in a constitutively active Notch 1 in T cell lymphoblastic leukaemia (T-ALL) [49]. This gene alteration is relatively rare occurring in <1% of T-ALL cases. However, a number of years later, sequencing studies identified *NOTCH1* activating mutations located in either the HD or PEST domains in some 50–60% of all patients [47], establishing Notch 1 as a bona fide oncogene in T-ALL. Similar *NOTCH1* and *NOTCH2* mutations have been seen in multiple B-cell malignancies including chronic lymphocytic leukaemia (CLL), mantle cell lymphoma (MCL), Hodgkin’s and Burkitt’s lymphomas, further supporting its role in these haematological malignancies [50,51,52].

Notch 3 and HES-1 were both shown to be overexpressed in T-ALL, with decreased Notch 3 expression showing an association with patient remission in the same study [53]. Despite the fact that Notch mutations are driving its overexpression in T-ALL, *NOTCH1* mutations are not predictive of prognosis and do not appear to be a useful biomarker aside from an observed association with improved early therapeutic response in T-ALL patients [54]. Overexpression of Notch signalling in the absence of gene alterations or mutations is also evident in other types of haematological cancer such as multiple myeloma (MM) [55] and acute myeloid leukaemia (AML) [56]. Unlike T-ALL, activating *NOTCH1* mutations in AML are rarely found; however, overexpression of Notch 1 and its ligands have been shown to be independent prognostic markers of overall patient survival [56,57,58,59]. In a recent AML study, Notch 3, 4 and Jagged-1 were associated with an adverse cytogeneic risk, Notch 2 and 3 expression were associated with increased relapse following induction therapy, Notch 4 and Jagged-2 were associated with increased relapse following allogeneic stem cell transplantation, while Notch 4, Jagged-2 and DLL-3 expression were associated with a poor OS in AML patients, further supporting the biomarker and therapeutic potential of Notch in this cancer [60].

### 2.3. Notch in Solid Malignancies: Driver Mutations and Biomarker Potential

#### 2.3.1. Breast Cancer

In the case of solid tumours, a role for Notch was first established in breast cancer. Notch signalling is involved in breast tumourigenesis, at least in part through the activation of oncogenic c-Myc [61], inhibition of p53-driven apoptosis [62], enhanced breast cancer stem cell (BCSC) self-renewal and proliferation [63], and the promotion of epithelial to mesenchymal transition (EMT) [64]. The majority of the research reported to date focuses on Notch 1 signalling as oncogenic pathways in breast cancer. Several studies have shown that Notch 1 and Jagged-1 expression are negatively associated with prognosis in breast cancer [65,66,67]. In a meta-analysis of 21 studies, including 3687 patients, Notch 1 was associated with progression from ductal carcinoma in situ (DCIS) to invasive cancer, and it was negatively associated with OS and progression-free survival (PFS) [67]. Using the cancer genome atlas (TCGA) breast cancer cohort (n = 956), a subset of patients with multiple hotspot mutations in the HD or PEST domain of Notch 1, 2 or 3, similar to that observed in T-ALL, were identified, rendering it constitutively active and/or resistant to degradation. About 50% of these patients were diagnosed with triple-negative breast cancer (TNBC). Mutant-Notch TNBC cases had higher gene expression of Notch 1 and other downstream proteins, and promisingly three mutant-Notch TNBC patient-derived xenograft (PDX) models were sensitive to Notch inhibition with a gamma secretase inhibitor (GSI). Notch 4 is significantly overexpressed in TNBC and HER2+ breast cancer compared to other breast cancer subtypes, and it was associated with a more aggressive clinical phenotype as well as poorer OS in luminal breast cancer patients [68]. Notch 3, which is critically involved with tumour-associated angiogenesis, appears to have a protumourigenic role in breast cancer, particularly highly angiogenic TNBC [69,70]. Notch 3 mutations, particularly in the PEST domain, have been linked to its overexpression in TNBC [71], suggesting its clinical relevance as suggested by others [72]. The less well-characterised role of Notch 2 in breast cancer is somewhat ambiguous, with several in vitro studies [73,74] suggesting a protumourigenic role and other in vivo studies reporting a tumour-suppressive role [75,76]. Stable knockdown of Notch 2 in TNBC xenografts demonstrated enhanced tumour growth, and notably Notch 1 was overexpressed in these tumours, suggesting an underlying mechanism of compensation [76]. In a clinical cohort of breast cancer samples, Notch 1 and Notch 2 were shown to have a reciprocal association with prognosis; high Notch 1 was associated with poor prognosis, while high Notch 2 was associated with better prognosis, suggesting a protective role of Notch 2 [77]. Similar to Notch 3, a number of PEST domain mutations have been identified in the Notch 2 receptor of TNBC patients leading to enhanced Notch signalling, indicative of oncogenesis [71]. Further work is required to definitively define the role of Notch receptors, in particular Notch 2, in the breast cancer setting.

#### 2.3.2. Lung Cancer

Similar to breast cancer, Notch signalling is an established oncogenic pathway in lung adenocarcinomas, the most common of which is nonsmall cell lung cancer (NSCLC). A genetic translocation of chromosome t (15;19) has been shown to be associated with overexpression of Notch 3 in NSCLC in approximately 40–50% of cases [78]. In another study, loss of NUMB expression, a negative regulator of Notch receptors was associated with 30% of NSCLC, and *NOTCH1* activating mutations were associated with 10% of cases [79]. Notably, the *NOTCH1* mutations observed occurred in the same hotspots as in T-ALL, i.e., the HD and PEST domains. A meta-analysis of 19 studies including 3663 patients indicated that high Notch 1 expression was associated with more aggressive disease (i.e., lymph node involvement and higher T-stage). Furthermore, in the same study overexpression of Notch 1/3, DLL-3 and the downstream gene target HES-1 were all associated with poor OS in NSCLC [80].

#### 2.3.3. Adenoid Cystic Carcinoma

Exome-sequencing of adenoid cystic carcinoma (ACC), a cancer of the salivary gland, has identified mutations in Notch pathway components in 11–29% of ACC patients [81]. In a subsequent expansion study, 14 of 102 (13.7%) ACC patients’ samples sequenced were found to harbour *NOTCH1* activating mutations in the T-ALL hotspots i.e., the HD and PEST domain [82]. Increased Notch pathway activity, as indicated by increased NICD proteins expression was reported in patients harbouring these gain-of-function *NOTCH1* mutations compared to patients with wild-type *NOTCH1*. Notably, 56% of the patient cohort showed the overexpression of NICD, indicating that aberrant Notch pathway signalling occurs in some patients independently of Notch 1 receptor genetic alterations. *NOTCH1* mutations were significantly associated with a more aggressive disease phenotype and although not an independent predictor of these measures, *NOTCH1* mutations were associated with decreased relapse-free survival and OS in comparison to *NOTCH1* wild-type patients [82]. NICD protein levels in the patients with suspected Notch-activating mutations were used as a surrogate marker to confirm a gain-of-function in the Notch-signalling pathway. In the same study, a Notch-receptor-targeted therapeutic antibody, brontictuzumab (OMP-52M51), showed significant and preferential antitumour efficacy in an ACC PDX model bearing a *NOTCH1* activating mutation. Furthermore, brontictuzumab showed a partial response in a double *NOTCH1*-mutant (HD and PEST domain) ACC patient who received two doses of the antibody therapy during a clinical trial. Unfortunately, the documented elevation of transaminases, suspected to be linked to brontictuzumab treatment, halted further treatment and the patient’s tumour soon progressed. Thus, Notch 1 is a newly emerging oncogenic driver of ACC.

#### 2.3.4. Colorectal Cancer

Recently, missense activating mutations in the Notch 1 and Notch 2 receptors have been identified in colorectal cancer; however, the functional relevance was not reported [83]. Notch 3 is overexpressed in a subset of colorectal cancer patients and induction of Notch 3 overexpression in colorectal cancer xenografts led to increased tumour formation [84]. A meta-analysis of 13 studies, including 3401 patients, reported a significant association between Notch 1 expression and colorectal cancer samples compared to noncancerous normal tissues, and clinical parameters were indicative of colorectal cancer invasion and metastasis (e.g., lymph node metastasis and depth of tumour infiltration in the surrounding tissue) [85].

#### 2.3.5. Other Solid Tumours

Aside from breast cancer, lung cancer, ACC and possibly colorectal cancer, cell-autonomous activating Notch mutations in solid tumours are less common than haematological cancers. However, upregulation of wild-type Notch receptors and ligands, and aberrant Notch signalling is frequently observed in various tumours including melanoma [86], gastric cancer [87], ovarian cancer [88], prostate cancer [89,90], pancreatic cancer [91,92], hepatocellular carcinoma [93,94,95], glioma [40,96,97] and rare tumours such as cholangiocarcinoma [98] and desmoid tumours [99]. Notch gene alterations may play a role in a subset of these cancer cases; however, such mutations are yet to be identified.

## 3. Notch Signalling in the Tumour Microenvironment: Interaction Between Cancer Cells and Other Cell Types

A tumour mass is not only composed of malignant cells, but it also contains blood vessels, immune cells, fibroblasts, signalling molecules and the extracellular matrix (ECM); collectively known as the tumour microenvironment (TME) [100]. Cells in the TME communicate via growth factors, cytokines and extracellular vesicles through paracrine signalling, and via membrane-type ligand/receptor pairs through juxtacrine signalling [101]. Much attention has been focused on the role of Notch signalling within the tumour cell; however, each compartment of the TME also expresses a variety of Notch ligands and receptors. Notch signalling is induced at cell-to-cell contact and, therefore, Notch juxtacrine signalling can regulate direct interactions between the TME and tumour cell [102].

Notch signalling is a major regulator of sprouting angiogenesis and the balance between DLL-4 and Jagged-1 has an impact on the tumour vascular architecture [103,104]. Tumour cells express Notch ligands and receptors that signal to endothelial cells (ECs) to activate angiogenesis. In a seminal study, co-injection of ECs with cancer cells overexpressing Jagged-1 led to an increase in microvessel density and tumour growth [105]. Mathematical modelling showed high Jagged-1 levels may lead to poorly perfused and chaotic angiogenesis, a hallmark of cancer, by destabilising the tip/stalk phenotype [106]. In endothelial-specific mouse mutants, modulation of endothelial Jagged-1 also regulated tumour vessel density and tumour vascular perfusion. Furthermore, endothelial Jagged-1 exerted an angiocrine function by activating Notch 2/HEY-1 in tumour cells promoting proliferation, survival and EMT [107]. Furthermore, endothelial-specific loss of DLL-4 resulted in tumour vessel regression along with a reduction in both EMT and cancer stem cells (CSCs) [108]. Interestingly, Notch activity is higher in tumour cells that are in close proximity to ECs. In ovarian tumour xenograft models, DLL-4 antibodies specific for the stroma reduced Notch signalling in the blood vessels and in tumour cells directly surrounding the blood vessels [109]. Furthermore, an EC-derived soluble form of Jagged-1 led to Notch activation in colorectal cancer cells in a paracrine manner [110]. In primary glioblastoma cultures, Notch ligands are expressed in ECs adjacent to Nestin and Notch receptor-positive cancer cells. Furthermore, coculture experiments with microvascular ECs and glioblastoma neurospheres show ECs promoted self-renewal in tumour cells. This indicates that activation of Notch in glioblastoma CSCs is driven by juxtacrine signalling with surrounding ECs to create a niche [111]. Overall, many aspects of the tumour vasculature are regulated by Notch signalling and, in addition, endothelial Notch ligands can send signals to tumour cells to increase the CSC phenotype leading to increased resistance and metastasis.

The immune infiltrates in the TME are major regulators of tumour progression and cancers must evade antitumour immune responses in order to progress [102]. Analysis of the TME has revealed a major subset of solid tumours show evidence of a T cell infiltration [112]. In T cell-infiltrated tumours, chemokines support the influx of CD8+ effector T cells, which subsequently become functional until they are inhibited by PD-L1, Treg cells and anergy [112]. Many studies have investigated the role of Notch signalling in T cell activation and effector functions. Activation of naive CD8+ T cells into effector CD8+ T cells requires Notch 1 that, in turn, induces expression of key effector molecules such as eomesodermin, perforin, and granzyme B through direct binding to their promoters [113]. Similarly, a conditional knockout of *NOTCH2* in CD8+ T cells showed that Notch 2 signalling is required for generating potent antitumour cytotoxic T cells [114]. Moreover, conditional deletion of Notch or pharmacological inhibition of Notch signalling diminishes the production of cytotoxic T cell effector molecules [113,115,116,117]. Naive T cells express full-length Notch receptors; however, T cells isolated from tumour-bearing mice have decreased expression of Notches 1–4 and a reduction in Notch target genes (*Deltex1, Hey1 and Hes1*) together suggesting T cells from tumours have repressed Notch signalling and decreased effector function [117,118,119]. This may be due to the recruitment of myeloid-derived suppressor cells (MDSC) to the tumour, which, in turn, block the expression of Notch in T cells [118]. On the other hand, overexpression of the Notch 1 intracellular domain renders CD8+ T cells resistant to the tolerogenic effect induced by MDSC [118]. A humanised anti-Jagged-1/2 blocking antibody prevented the accumulation and tolerogenic activity of MDSCs in tumour-bearing mice and increased the infiltration of reactive CD8+ T cells [120]. Another study reported that mechanism of escape from T cell immunity is caused by a reduction of DLL-1 in bone marrow precursors, which results in suppressed T cell function [121]. Indeed, systemic DLL-1 administration in a murine lung cancer model increased T cell infiltration into tumours, as well as, elevating memory T cells, decreasing Treg cells and limiting tumour vascularisation [122]. The role of Notch signalling in the modulation of CD8+ T cell activity may be context-dependent. In patients with colorectal carcinoma, inhibition of Notch signalling promoted the cytotoxicity of CD8+ T cells by decreasing PD-1 expression [123]. In addition, a study in lung adenocarcinoma patients showed inhibition of Notch in peripheral and lung resident CD8+ T cells augments their cytotoxicity and promotes interferon-γ production [124].

Macrophages are one of the major tumour infiltrating immune cells and they are advantageous for tumour growth and metastasis. Similarly to T cells, Notch signalling is involved in macrophage activation and effector functions [125]. Tumour-associated macrophages (TAMs) from mammary mouse tumours require the transcriptional regulator of Notch signalling, RBP-J, for terminal differentiation [126]. TAMs participate in immune responses in a polarised manner: classic M1 macrophages produce IL-12 to promote tumouricidal responses, whereas M2 macrophages produce IL-10 to induce tumour progression [127]. Notch signalling plays critical roles in the determination of M1 versus M2 polarisation of macrophages, and compromised Notch pathway activation leads to the expansion of M2-like TAMs [128]. Furthermore, the Jagged-1-Notch pathway is elevated in aromatase inhibitor-resistant breast cancer cells resulting in the differentiation towards M2 TAMs, which contributes to the acquisition of resistance [129]. On the other hand, conditional activation of the Notch 1 ICD abrogated TAM function leading to tumour regression in syngeneic mouse models [130]. It is clear that Notch signalling plays an important role in the immune infiltrate signalling within the TME. The contradictory reports on the role of Notch in T cells and TAMs imply that the signalling is dependent upon the tumour type and/or components of the TME. In addition, different Notch ligands may activate Notch in a variety of ways and this may impact the phenotype of the immune infiltrate. Targeting Notch with pan inhibitors may reinforce the immunosuppressed TME in certain tumours and therefore evaluation of Notch inhibitors must take into consideration the subpopulation of cells within the TME. In addition, given the importance of Notch in the immune infiltrate, syngeneic graft or genetically engineered mouse models should be favoured over xenografts models when evaluating Notch inhibitors in vivo.

A heterogeneous population of fibroblast-like cells, termed cancer-associated fibroblasts (CAFs), are important contributors to tumourigenesis [131,132]. Hu et al. showed mice with a mesenchymal cell-specific deletion of CSL/RBP-J, a key Notch effector, exhibit spontaneous keratinocyte tumours followed by dermal atrophy and inflammation. The CSL-deficient dermal fibroblasts promote tumour cell proliferation through upregulation of inflammatory cytokines and matrix remodelling enzymes [133]. Moreover, in human skin samples, stromal areas adjacent to multifocal premalignant actinic keratosis lesions exhibited decreased Notch/RBP-J indicating a role for Notch signalling in adjacent fibroblasts to prevent tumour formation [133]. In a prostate cancer model, Jagged-1 upregulation did not affect tumourigenesis but promoted an increase in the percentage of CAFs, which led to the development of a reactive stromal microenvironment [134]. Similarly, colon cancer cells induced the transformation of bone marrow mesenchymal stem/stromal cells to CAFs via Notch-Jagged-1 signalling [135]. In breast cancer, CAFs have been shown to act in a paracrine manner through secretion of inflammatory modulators such as IL-6, inducing Notch activation in cancer cells and thus promoting the stem cell phenotype and resistance [136,137]. Furthermore, in hepatocellular carcinoma (HCC), CAFs secrete high levels of IL-6, which promoted stem cell-like properties in HCC cells by activating Notch 1 signalling through STAT3 Tyr705 phosphorylation [138]. In addition, CAFs isolated from HCC tissues can promote the CSC phenotype through Notch 3 signalling [139]. Adipocytes also play an active role in the TME and cancer-associated adipocytes have smaller lipid droplets and a more fibroblast-like phenotype, compared to normal adipocytes [140]. When preadipocytes were cocultured with breast cancer cells, there was enhanced CAF marker expression and IL-6 secretion, which promotes tumourigenesis; although the activation of Notch was not investigated [141]. RNA within exosomes released from stromal cells stimulated STAT1-dependent antiviral response signalling in breast cancer cells. The STAT1 facilitates transcriptional responses to Notch 3 and expands the CSC subpopulation and this was abrogated by a GSI [142]. Loss of Notch 1 in CAFs increased the stemness phenotype of melanoma cells and this was correlated with tumour aggressiveness and metastasis [143].

Overall, a clearer understanding of the roles of Notch signalling in the TME is required for a better interpretation of the efficacy and toxicity of Notch targeting therapies. The use of an antibody directed nanoparticles to specifically inhibit Notch signalling in a specific cell population are in development and may offer a more efficient way to target Notch signalling in cancer [102].

## 4. Notch Crosstalk with Other Signalling Pathways and Therapy-Resistance Mechanisms

Given that Notch signalling is frequently overexpressed in cancer and has a key role in a diverse range of cellular processes, it is not surprising that Notch displays diverse crosstalk with many other oncogenic signalling pathways such as developmental signals (Hedgehog and WNT), growth factors, steroids, oncogenic kinases and transcription factors. Increasing evidence suggests Notch signalling is associated with treatment resistance to molecular therapies targeting other oncogenic pathways including HER2 (e.g., trastuzumab), EGFR (e.g., gefitinib), proangiogenic VEGF signalling (e.g., brivanib and sorafenib) and steroid signalling (e.g., tamoxifen and enzalutamide) [144,145,146,147,148,149,150] or with resistance to cytotoxic chemotherapeutic agents and radiation treatment [151,152]. In recent years, dual or multitargeted therapy approaches have become an attractive treatment strategy to combat resistance. There is, therefore, a rationale for attacking the cancer from multiple nodes, resulting in synergistic effects, perturbing the upregulation of compensatory protumourigenic pathways such as Notch signalling. The combination of Notch signalling inhibitors with a second molecularly targeted drug is a research area in its infancy. However, initial preclinical studies show huge potential. Examples of known interactions of Notch with other key protumourigenic pathways are discussed in this section.

### 4.1. Interaction with Tyrosine Kinase Receptors; HER2 and EGFR

The Notch pathway is implicated in targeted treatment resistance in ErbB2/HER2 positive breast cancer, which is potentially attributed to the coregulation between Notch 1 and ErbB2/HER2 signalling. Following treatment with trastuzumab or a dual epidermal growth factor receptor (EGFR)/ErbB2 tyrosine kinase inhibitor (TKI; i.e., lapatanib), in a range of HER2 positive breast cancer cell lines, HER2 was downregulated, while the Notch activity was upregulated. Trastuzumab treatment, in particular, increased nuclear accumulation and activation of Notch 1 ICD, which led to upregulation of the Notch 1 target genes including *HEY1*, *HES5, HES1* and *DELTEX1* [144]. In the same study, a combination of a GSI and trastuzumab abrogated resistance and enhanced apoptosis of breast cancer cells by 20–30%. Interestingly, both trastuzumab sensitive and resistant cancer cell growth was completely inhibited when Notch 1 was knocked down using siRNA [144]. In light of these results, it is likely that when HER2 is overexpressed, cancer cells rely on HER2 protein for survival whereas when HER2 is downregulated, as part of a compensatory mechanism, the Notch pathway is reactivated to maintain cancer cell survival. Similar results were demonstrated in vivo, where Notch 1 activation was responsible for tumour dormancy, which led to treatment resistance and drastically faster recurrence of HER2/neu-induced mammary tumours [153]. The same study demonstrated in a meta-analysis of 4463 patients, that elevated Notch activity was associated with reduced recurrence-free survival [153]. In support of this, overexpression of Notch 1 and Jagged-1 was also associated with poor prognosis for all types of breast cancer, including HER2+ cancer [65]. Overall, these findings suggest that a combination of a Notch 1-targeted therapeutic agent with other HER2-targeted therapies, such as trastuzumab or lapatanib, would be beneficial in abrogating HER2 treatment resistance.

EGFR is one of the most commonly mutated and overexpressed genes in NSCLC. The survival time of lung cancer patients bearing common EGFR mutations (Exon 19 deletion or L858R point mutation) has been markedly improved with the FDA approval of first-generation EGFR tyrosine kinase inhibitors (TKIs), gefitinib and erlotinib [154]. While most EGFR-mutant lung cancer patients respond well to begin with, the development of resistance is inevitable. In 60% of cases, this is due to further acquisition of ”gate-keeper” mutations in EGFR, namely T790M [145]. Osimertinib, a T790M-specific TKI, has been FDA approved for the treatment of metastatic NSCLC harbouring EGFR T790M mutations, whose disease has progressed on or after EGFR TKI therapy [155]. Similarly, while initial responses were good, resistance soon developed as a result of further EGFR mutations or the emergence of EGFR-independent resistance mechanisms, including Notch signalling. As discussed previously, Notch signalling is associated with poor prognosis in NSCLC (Section 2.3.2). Researchers have shown that Notch 1 is overexpressed in a lung cancer cell line model of acquired resistance to erlotinib and drives an EMT-like phenotype [156]. Others have shown that Notch and Akt-specific inhibitors can reverse resistance to erlotinib in TKI-resistant lung cancer cells and that Notch/Akt driven resistance is associated with upregulation of the oncogenic miR-233 [157]. Importantly, in xenografts of the same TKI-resistant lung cancer cells, the dual treatment with erlotinib and the Notch inhibitor (DAPT) showed significant antitumour efficacy. Another study showed that while inhibition of EGFR led to significant cell death in EGFR-mutant lung cancer cells, it increased stemness properties, e.g., higher ALDH+ population and increased clonogenicity, which was likely driven by an increase in Notch 3 expression [158]. Combined inhibition of EGFR and Notch 3 using pan-Notch GSIs was able to reverse the stem-like phenotype [158]. Subsequent work showed that EGFR TKI activation of Notch 3 in EGFR-mutant lung cancer cells is accompanied by stabilisation and activation of β-catenin [159].

Beyond lung adenocarcinoma, Notch 3-specific depletion, via siRNA transfection, has been shown to sensitise TNBC cells to gefitinib treatment [160]. Recent work has shown that NSCLC PDX models harbouring secondary EGFR “gate-keeper” mutations (e.g., EGFRT790M/L858R) that drive resistance to gefitinib and osimertinib can be resensitised to these drugs by including a Notch inhibitor as part of the treatment regimen [145]. In these PDX models, as expected, EGFR TKIs had no effect on tumour growth, whereas GSI dibenzazepine (DBZ) monotherapy inhibited tumour growth, and dual TKI/GSI treatment completely blocked tumour growth. A decreased expression of the Notch target gene and transcription repressor, HES-1, was associated with combined TKI/GSI treatment but not with either treatment alone, and it was shown to be STAT3-dependent.

Clinically relevant GSI, nirogacestat, showed similar activity in combination with gefitinib in an EGFR T790M lung adenocarcinoma cell line xenograft model, providing further rationale for this combined therapeutic strategy in patients with EGFR “gate-keeper” mutations [145]. Another clinical GSI, RO4929097, in combination with erlotinib was investigated in NSCLC patients in a Phase I/II trial (NCT01193881; Table 1). While Roche ceased all production and development of RO4929097 due to a lack of study endpoints, resulting in the early termination of a number of trials including NCT01193881, the results obtained indicate the combination was safe and tolerable in lung cancer patients [161].

### 4.2. Phosphatidylinositol 3-Kinase (PI3K)/Akt and Notch Signalling

Simultaneous activation of the PI3K/Akt and Notch pathways has been shown to induce tumourigenesis and tumour progression [201,202]. Using a Drosophila fly model, a number of selective compounds have been identified that concurrently target PI3K/Akt and Notch, exhibiting anti-inflammatory properties via inhibition of nitric oxide synthase (NOS) and lipoxygenase (LOX), leading to tumour inhibition [202]. In T-ALL cells-derived from patients, Notch via HES-1 was able to suppress PTEN, which inhibits the prosurvival PI3K/Akt pathway [203]. In addition, blocking Notch 1 activity has been shown to upregulate *PTEN* gene expression in T-ALL cells via *HES1* downregulation. Indirectly, Notch is also able to enhance the expression of IL-7 [204] and insulin growth factor-1 receptors [205]; known activators of the PI3K/Akt pathway [206]. Another target of Notch is the tumour suppression gene, *p53*, which is negatively regulated by the Akt substrate, Mdm2 [207]. In fact, in a number of murine T-ALL models, downregulation of Notch upregulated *p53*, led to tumour inhibition [208]. As discussed previously, approximately 50% of T-ALL tumours have gain-of-function *NOTCH1* mutations [47], therefore, it is critical for this patient group that therapeutic agents capable of inhibiting Notch 1 are developed. In relation to Notch 4, four distinct Akt phosphorylation binding sites within Notch 4 ICD were identified in breast cancer in vitro and in vivo models [209]. Interestingly, once phosphorylated, these binding sites within Notch 4 ICD were utilised by 14-3-3 regulatory proteins, hence preventing nuclear translocation of Notch 4 ICD and inhibiting Notch signalling [209]. The cooperation of Notch and PTEN/PI3K/Akt signalling contributes to tumourigenesis in a number of solid malignancies. As discussed previously, Notch 1 is associated with HER2+ breast cancer resistance to trastuzumab (Section 4.1). A recent study has suggested that Notch 1 suppression of PTEN enhances cell proliferation and stem cell survival in treatment-resistant HER2+ breast cancer, via upregulation of ERK1/2 [210]. Increased Notch 1 activity has been shown in a PTEN loss-of-function preclinical model of prostate cancer and treatment with GSIs can effectively elicit tumour growth arrest [211]. Further insight into the mechanism linking PTEN loss to ligand-independent Notch 1 signalling suggests that upregulation of ADAM 17 expression in the absence of PTEN is responsible for enhanced cleavage and activation of Notch 1 [211]. Similar to T-ALL, Notch inhibition with GSIs in glioma cancer cells has been shown to induce PTEN and suppress PI3K signalling, via downregulation of the transcriptional regulator, HES-1. Furthermore, glioma cancer cells with PTEN loss-of-function mutations are less sensitive to GSI treatment than cells bearing wild-type PTEN [212]. The authors suggest that GSI resistance involves an oncogenic switch from Notch to PI3K/Akt signalling and the combination of GSI and PI3K inhibitor in PTEN mutant glioma potentially has synergistic antitumour efficacy. Additionally, the combination of Notch and PI3K/Akt inhibition demonstrated synergistic antitumour activity in gastric cancer and reduced metastasis in vivo compared to either therapy alone [213]. This data suggests that the complex coregulatory interplay between the Notch and PI3K/Akt pathways needs to be further elucidated in order to optimise combinational treatment regimens that can selectively and synergistically inhibit both of these tumourigenic pathways.

### 4.3. Notch in Steroid Hormone Therapy-Resistant Cancer

Steroid receptors are a family of nuclear receptors, including oestrogen receptors (ER), progesterone receptors (PR), androgen receptors (AR) and glucocorticoid receptors (GRs). ER/PRs and AR drive cell growth, proliferation and metastasis in malignancies such as breast cancer and prostate cancer, respectively, thus therapeutic interventions focusing on limiting oestrogen, progesterone or androgen production, or preventing steroid binding to their respective receptors, could be beneficial. In contrast, glucocorticoids are not considered oncogenic and instead elicit antiproliferative and proapoptotic effects in lymphoid tissues; thus, they are often used as part of treatment strategies to manage lymphatic cancers.

The majority of breast cancer patients are ER+, meaning that the tumours are dependent on oestrogen for growth [214]. Tamoxifen or fulvestrant are competitive ER antagonists routinely used in the treatment of ER+ cancers; however, resistance to endocrine therapy and relapse often occurs. The overexpression of Notch signalling is associated with the use of antioestrogen therapy and treatment resistance in breast cancer cells [215,216,217]. In tamoxifen-resistant ER+ breast cancer patients, Notch 1 and oestrogen receptor-1 (ESR1) showed reciprocal regulation as well as Notch 1 overexpression; potentially indicating Notch 1′s involvement in endocrine resistance [218]. In an additional study, treatment of ER+ breast cancer with tamoxifen or fulvestrant resulted in an increased BCSC population, via upregulation of Jagged-1 and Notch 4 signalling [148]. Furthermore, high pretreatment levels of Notch 4 and ALDH1 (a marker of stemness) were associated with poorer outcomes in ER+ patients treated with antioestrogen therapy. Notch inhibition with a GSI in vivo reduces BCSC activity in long-term acquired resistant ER+ PDX tumours, suggesting resistance can be combatted by combining antioestrogens with anti-Notch therapies [148].

Androgen deprivation therapy (ADT) with the use of AR antagonists such as enzalutamide is standardly used in the treatment of late-stage and metastatic prostate cancer and similar to ER antagonists, it is associated with a short response period and subsequent drug resistance [90,150]. A recent study has indicated that Notch 2 is overexpressed in enzalutamide-resistant prostate cancer patients, while cleaved or activated Notch 1 and HES-1 levels were increased in enzalutamide-resistant cell line models [219]. Treatment of enzalutamide-resistant prostate cancer cells with the GSI, PF-03084014, or an alternative specific gene knockdown of Notch 1, resensitised cells to enzalutamide treatment, while the drug combination showed efficacy in xenograft models [90]. ADT has been shown to increase Notch 3 expression in prostate cancer cells while activation of any of the four NICDs increases resistance to ADT in androgen-dependent prostate cancer cells [150]. In the same study, treatment with the GSI, DAPT can enhance the efficacy of ADT, indicating its potential as an adjuvant therapy with ADT in prostate cancer. The synergy of Notch inhibition and ADT has been demonstrated in ERG-driven prostate cancer in combination with both AR antagonists such as enzalutamide and inhibitors of androgen synthesis such as abiraterone [149].

Notch has been shown to interact with GR signalling pathways. Glucocorticoid drugs, such as dexamethasone are commonly part of the treatment schedule for T-ALL patients. Binding to the GR, NR3C1, leads to subsequent upregulation of a range of proapoptotic proteins including Bcl-2 family members such as BIM, triggering cell cycle arrest and apoptosis in leukaemia lymphoblasts [212]. The importance of glucocorticoid treatment in T-ALL is highlighted by the increased incidence of disease recurrence and poor clinical outcomes associated with acquired steroid drug resistance [212]. GSIs have been shown to reverse glucocorticoid resistance in T-ALL cell lines and primary lymphoblasts [220]. In preclinical studies, PF-03084014 in combination with dexamethasone was shown to have synergistic antileukemic effects in vitro, enhancing the proglucocorticoid gene signature, including BIM [221]. In vivo, the combination of drugs significantly suppressed tumour growth compared to either drug alone. Critically, the undesired gastrointestinal (GI) on-target toxicity associated with GSI systemic Notch inhibition (see Section 5.1 for more details) was reversed by the cotreatment with glucocorticoid in vivo [221]. A recent study in osteoblasts has indicated that glucocorticoids can suppress the transcription of Notch target genes (e.g., the HEY family of transcription repressors) through a mechanism that is independent of Notch activity and NICD/RBP-J/DNA interaction [222]. Thus, it is possible that an indirect increase in Notch-regulated transcription of HES and HEY family genes in response to glucocorticoid-induced suppression of these very same genes, give rise to the steroid drug resistance observed in T-ALL. Given the combination of glucocorticoids and GSIs can mitigate GI toxicity without hampering the antitumour effects of GSIs, there is a scope for the use of this drug combination in other Notch-driven cancers beyond T-ALL. LY3039478 is a potent GSI that has been shown to effectively inhibit Notch activity in preclinical studies [223,224] and in clinical trials [175] (see Section 5.1.2 for further detail). A Phase Ib trial to evaluate the safety of LY3039478 in combination with dexamethasone in patients with T-ALL or T-LBL (T cell lymphoblastic lymphoma) was completed in 2019, and while no patient had a complete remission, a dose of 75 mg LY3039478 and 24 mg dexamethasone was shown to have a good safety profile for further clinical evaluation (NCT02518113).

### 4.4. The WNT, Sonic Hedgehog and Notch Pathway Crosstalk

Other tumourigenic pathways that interact with Notch include WNT and Sonic Hedgehog. The WNT pathway transduces signals both as a canonical and noncanonical pathway, similar to the Notch pathway. The canonical pathway signals through Frizzed (Fzd) and Lrp5/6 receptors to WNT/β-catenin and WNT/Stop, whereas the noncanonical signalling starts with the Fzd and/or Ror1/Ror2/Ryk receptors to the WNT/Pcp and WNT/Rtk and WNT/Ca^2+^ signalling cascades [225,226]. Both signalling cascades in cancer are involved in regulating the development and expansion of CSCs [227]. Without WNT, β-catenin in the cytosol undergoes phosphorylation by GSK-3β and CK1, enters the destruction complex, which is then tagged by the E3 ligase, β-TrCP, for proteasomal degradation [228]. Increased expression of β-catenin has been reported in basal breast cancer and is associated with poor prognosis, suggesting that inhibition of WNT could be a possible therapeutic strategy for reducing β-catenin expression and hence tumour progression [229]. Overall, aberrant WNT signalling has been linked to haematopoietic malignancies, and development and recurrence in a number of solid cancers [230].

Sonic Hedgehog also has a critical role in the development and CSC signalling, and it is closely interconnected with the WNT pathway. Once activated, it attaches to Patch receptors, which leads to activation of the Smoothened receptors (Smo), and downstream transcription of glioma-associated oncogenes homologs (*GLI1/2/3*) [231]. Crosstalk between the WNT, Sonic Hedgehog and Notch pathways has been demonstrated although not fully elucidated. These three pathways are highly abundant in CSCs and closely associated with tumourigenesis and tumour recurrence [227]. β-catenin is capable of activating Notch signalling by increasing the expression of Jagged-1, a ligand of the Notch 1 receptor [230]. Similarly, β-catenin also interacts with Notch 1 receptor, decreasing Notch 1 ubiquitination and hence stimulating activation of HES-1, which has been linked to tumourigenesis. One of the key partners in crosstalk signalling between the WNT and Notch pathways is GSK-3β. This enzyme is responsible for phosphorylation of Notch 1 ICD, its nuclear translocation and transcriptional activation of downstream targets [232]. On the other hand, crosstalk between the Sonic Hedgehog and Notch pathways is less well-understood. However, Notch has been implicated as one of the key mechanisms for intracellular transport to the primary cilia, which is an important component of the Sonic Hedgehog pathway [233]. Additionally, a loss-of-function mutation in *PATCH1* gene, led to the development of Notch 1-induced T-ALL suggesting that Sonic Hedgehog drives, a well-known mutation in this malignancy [234].

### 4.5. Nuclear Factor κB (NFκB) and Notch Pathway Interaction

NFkB consists of a family of transcription factors that play critical roles in the immune system and inflammatory responses in a number of diseases including cancer [235]. The multilayered crosstalk between the Notch and NFkB pathways is particularly important for Tregs residing within the TME [236]. As described above, Tregs are immune cells that suppress the unwanted immune reactions and infiltration of Tregs within tumours has been associated with the lower ratio of cytotoxic CD8+ T cells to Tregs [237], tumour progression [238] and poorer prognosis [239]. In T-ALL, the NFkB pathway can be activated extracellularly by pre-T cell constitutive Notch 3 activation [53]. Similarly, Notch 1-mediated crosstalk with NFkB is also present in T cell leukaemia, where Notch 1 regulates the expression and binds to NFkB subunits (e.g., Relb and Nfkb2), hence regulating downstream transcriptional changes [240]. An example of interaction between Notch and NFkB starts with Notch 1 forming a complex with IKKα, which translocates to the nucleus, as demonstrated using cervical cancer cells [33]. In prostate cancer, Notch 4′s effects on prostate cancer cell proliferation, growth, EMT, migration and invasion were shown to be dependent on the NFkB pathway. Therefore, when Notch 4 was silenced, this led to inhibition of important hallmarks of prostate cancer cells, which was shown to be dependent on the NFkB pathway [89]. Both Notch and NFkB are essential in maintaining the survival of CSCs [241]; however, the interplay between these two pathways in the context of CSC inhibition is not well-understood. While both NFkB and Notch can target CSC surface markers, NFkB is capable of inducing CSC apoptosis whereas Notch is implicated in stem cell fate and differentiation [242].

### 4.6. The Notch Pathway and Regulation of Epithelial to Mesenchymal Transition

EMT is one of the key processes driving tumour progression and metastasis, and it is characterised by transdifferentiation of adhesive epithelial-like cancer cells to more motile/metastatic mesenchymal-like cancer cells. It is a dynamic process that facilitates invasion and metastasis of cancer cells to secondary tumour sites. Once homed, these cells undergo the reverse process where mesenchymal-like cells are converted back to epithelial-like cells, which leads to the formation of secondary tumours through mesenchymal to epithelial transition (MET) [64]. EMT is transcriptionally controlled, to varying degrees, by a number of genes including *CDH1* (E-cadherin)*, SNAI1/2, ZEB1/2*, *TWIST1/2, FOXC1/2, TCF3 and GSC* (Goosecoid) genes [243]. In fact, E-cadherin appears to be the main regulator of several EMT-associated transcription factors such as SNAIL 1, SLUG, ZEB 1/2, TWIST, FOXC 1/2, TCF and Goosecoid [244]. Other pathways involved in the regulation of the EMT-inducing transcription factors, particularly in breast cancer, include the WNT, Hedgehog, Notch and extracellular integrin pathways [245]. Cancer cells, which have undergone EMT, typically display the following profile of markers: low E-cadherin, high vimentin and high N-cadherin protein expression [246]. Since epithelial cells lose their adherent properties as they become more migratory, the extracellular matrix needs preadjusting to accommodate newly formed mesenchymal cells. As a result, extracellular matrix proteins such as fibronectin, collagens and proteases become upregulated [247].

The Notch pathway activates EMT through the NF-ĸB pathway leading to SNAIL 1 activation or by regulating TGF-β signals involved in Notch activation, as part of the feedback loop [64,248]. Hypoxia also plays a role in Notch-mediated EMT regulation through HIF-1α and HIF-1β, which activate SNAIL 1and SLUG and downregulate E-cadherin [64,245].

Notch 1 pathways in particular seem to play an important role in EMT. Jagged-1, through upregulation of Notch 1 ICD mediates EMT activation by downregulating E-cadherin via SLUG [249,250]. Conversely, when Notch 1 was silenced, it led to inhibition of cell migration and invasion, as well as tumour growth, by abrogating EMT, which was reversed when SLUG was overexpressed [251]. Overall, three potential mechanisms of Notch-mediated activation of EMT have been proposed: (1) cytokine (IL-6)-mediated activation of Notch 2 via JAK/STAT3 and NFkB signalling [252], (2) Notch 1-mediated activation of the PI3K/Akt pathway, STAT3 and protein phosphatase 2A [253], and (3) hypoxia-mediated activation of HES-1 and HEY-1 [254], as summarised by Kar et al. [64].

### 4.7. The Notch Pathway and Cell-to-Cell Adhesion

Notch signalling can modulate cell behaviour and promote cell adhesion processes. As discussed in the previous section the EMT process involves Notch signalling and promotes the detachment of a cancer cell from neighbouring cells promoting invasion through the basement membrane. Notch signalling has been reported to promote intravasation, i.e., the process by which a migrating cancer cell crosses the endothelial cell barrier of tumour vessels and enters the circulation. Notch receptor activation on endothelial cells occurs via an interaction with DLL-4 and Jagged-1 ligands expressed on tumour cells [255]. This promotes endothelial cell senescence and subsequent weakening of endothelial cell tight junctions, and the induction of senescence-associated proinflammatory cytokines and the adhesion molecule vascular adhesion molecule 1 (VCAM1). Overall, this facilitates the transmigration of cancer cells (via an interaction of endothelial cell VCAM1 with cancer cell α4-integrin) and immune cells into the circulation, and homing to distant metastatic sites. Thus, Notch signalling can induce cancer cell-endothelial cell adhesion and promote metastasis.

While Notch receptors and ligands are generally described as signalling molecules, early fly studies and a more recent mammalian study demonstrate that Notch receptors can also act as functional cell adhesion molecules on the cell surface [256,257]. Due to the transient nature of the adhesion observed between Notch receptors and ligands in drosophila, it was not seen as an important source of physical cell-to-cell contact [256]. This was further supported by the established immediate cleavage of Notch receptors following ligand interaction during a signalling event. However, a more recent mammalian study involving mouse mast cells and stromal cells reported a prolonged cell-to-cell adhesion role of the Notch receptor-ligand that did not involve Notch signalling. It remains to be elucidated what the physiological functions of the Notch receptor-ligand is as an adhesion molecule despite its inevitable cleavage to release the NICD signalling molecule.

### 4.8. Notch Signalling in Cancer Stem Cell Renewal and Differentiation

Notch is a critical pathway is the maintenance of normal stem cells and CSCs in solid tumours such as glioblastoma [258], ovarian cancer [259], and breast cancer [260]. Nevertheless, aberrant activation of Notch appears to be predominantly present in CSCs. CSCs encompass a small percentage of cancer cells within the tumour; however, these cells are highly tumourigenic and resistant to a number of anticancer treatments [261]. For example, in breast cancer, CSCs do not respond to chemo-, radiotherapy or tamoxifen treatment, whereas therapeutic agents such as GSIs or AD-01/ALM201 targeting self-renewal and/or differentiation pathways were effective at inhibiting CSCs [262,263]. AD-01 and ALM201 were capable of inhibiting DLL-4 and Notch 4 [262]. DLL-4 antibodies have also been utilised previously to target CSCs in colon tumour xenografts, which led to a reduction in tumour growth, and the delay in tumour initiation and tumour recurrence [264]. The mechanism of this effect comprises differentiation of CSCs into more mature/treatment sensitive cells, which was demonstrated by downregulation of *HES1* target gene responsible for maintaining cells in an undifferentiated state as well as the upregulation in *Atoh1* indicative of differentiation of colon cells into mucin-producing goblet cells [264].

The roles of Notch 1 and Notch 4 in breast CSCs were elegantly demonstrated by Harrison et al. [260] suggesting that Notch 4 signalling was more pertinent for CSC maintenance than Notch 1. Interesting, GSIs were more effective at inhibiting Notch 1 expression, which was four-fold lower in CSCs, whereas there was no effect on Notch 4, which was eight-fold higher within CSCs; high Notch activity therefore, led to initiation and maintenance of tumours. Following the knockdown of Notch 4 or Notch 1, the more efficient inhibition of mammosphere formation in vitro and tumour initiation in vivo was observed in the Notch 4 knockdown model; results representative of the effects on BCSCs. Furthermore, Notch 4 exhibited increased activation when Notch 1 was knocked down, indicating a compensatory mechanism. Therefore, while the Notch pathway inhibitors appear to target the self-renewal abilities of the CSCs, targeting GS enzyme in breast cancer might not be the most optimal strategy in terms of CSC inhibition.

Interestingly, in ovarian cancer, there is evidence that Notch is activated in cancer cells, which are close to the TME and that the small vessels expressing Notch ligands such as Jagged-1 and DLL-1/3/4 are key in regulating Notch signalling in cancer cells [109]. Similarly, in colon cancer, soluble Jagged-1 released from endothelial cells by ADAM metalloproteases within the TME was shown to activate Notch and increase the abundance of CSCs within cancer cell population [110]. Therefore, the most optimal strategy for targeting different components of the Notch pathways in order to reduce or inhibit tumourigenic and treatment-resistant CSC, still remains a challenge, both in terms of effectiveness and safety, as a number of Notch inhibitors enter or continue in various clinical trials (Table 1).

### 4.9. Notch in Resistance to Chemotherapy and Radiation Therapy

The use of a molecularly targeted therapy in combination with chemo- or radiation therapy has become a popular avenue of therapeutic exploration. A number of targeted therapies are capable of inhibiting the drug-resistant subpopulation of self-renewing CSCs therefore sensitising more of the tumour to standard cytotoxic therapies and prolonging antitumour responses. It is widely accepted that CSCs have in-built mechanisms of resistance to chemo/radiation therapy such as aberrant Notch signalling as described above, compared to the differentiated, non-CSCs of the tumour bulk, allowing them to reinitiate and repopulate the tumour after treatment. As discussed previously, Notch signalling is critically involved in CSC renewal and differentiation (Section 4.8), making Notch inhibitors an attractive combination treatment strategy with standard cytotoxic therapy. Chemotherapy induces Notch activation and inhibition of Notch sensitises cancer cells to chemotherapy in multiple studies across different cancer types including T-ALL [265], breast cancer [266] ovarian cancer [259], glioma [267], hepatocellular carcinoma [268], prostate cancer [269,270,271], colorectal cancer [272,273], lung cancer [151,274,275] and osteosarcoma [276]. Similar results have also indicated the role of aberrant Notch signalling in radiation therapy resistance [249,267,277]. Combined treatment of clinically relevant GSI AL101 (formerly BMS906024) and paclitaxel or cisplatin demonstrated synergetic tumour inhibition compared to either drug alone in adenocarcinoma cell lines and PDX models, especially those with wild-type KRAS and BRAF [151]. Notch 3 has been shown to be upregulated following radiation in NSCLC cell lines and Notch inhibition with a GSI, which radiosensitises the cells [152]. Subsequent mechanistic work indicated that HIF-1α can further enhance radiation-induced Notch 3 activation under hypoxic conditions. The combination of HIF-1α inhibitor, YC-1 and Notch GSI had increased radiosensitivity compared to either agent alone in NSCLC xenografts [278]. Given that most lung cancer patients are treated with a combination of chemotherapy and radiation therapy, a recent study investigated the role of Notch signalling in combined chemoradiation therapy in NSCLC. Results suggest an increased sensitisation of 3D NSCLC tumour spheroids in vitro when adding GSI to chemotherapeutic crizotinib alone or in triple combination with crizotinib and radiation [279]. In hepatocellular carcinoma, Notch 3 activation specifically was shown to contribute to doxorubicin resistance through a mechanism involving p53 regulation and this effect could be abrogated by silencing Notch 3 [268]. Numerous studies have shown that activation of Notch signalling enhances chemoresistance in prostate cancer and use of Notch antagonists can greatly resensitise the tumour cells to standard chemotherapies such as docetaxel and paclitaxel in vitro and in vivo [270,271,280]. The growing numbers of studies in multiple cancers provide strong support and rationale for the combination of Notch inhibitors and standard cytotoxic therapies in future clinical trials, as a strategy to overcome the therapy resistance frequently observed in the clinic. A number of clinical trials have already indicated the safety of such a combination of therapies (see Section 5 and Table 1).

## 5. Inhibiting the Notch Pathway with Molecular-Targeted Therapies

Given frequently observed aberrant expression of Notch in cancer, its well-established roles in tumourigenesis and metastasis as well as reported biomarker potential in several cancers, there has been a growing interest in therapeutically targeting Notch via single agents or using a multimolecular-targeted approach. As a result of extensive research on the regulation of Notch signalling, three strategies of therapeutically inhibiting Notch have emerged: (1) inhibiting the proteolytic cleavage/activation of the receptor(s) using small molecular GSIs, (2) inhibiting the initial ligand–receptor interaction using neutralising monoclonal antibodies or receptor decoys and (3) suppressing the transcriptional coactivator role of Notch in the nucleus (see Figure 1).

### 5.1. Inhibition of Gamma Secretase

Gamma secretase is a membrane-bound aspartyl protease complex consisting of a catalytic subunit named Presenilin (PSEN1 and PSEN2) and three others subunits including Nicastrin, APH-1 (APH1A and APH1B) and PEN-2 [281]. Aside from the cleavage of four known Notch receptors, the gamma secretase complex is also involved in proteolytic cleavage of over 90 other membrane-bound protein substrates including ErbB4, E-cadherin, CD44 and amyloid precursor protein (APP) indicating the varied physiological role of this enzyme [282]. The sequential cleavage of APP releases β-amyloid peptides that accumulate as the insoluble amyloid plaques associated with Alzheimer’s disease [283]. Small-molecule GSIs were first developed and used for the treatment of Alzheimer’s disease, but given the comparable involvement of gamma secretases in Notch activation, some of these drugs are being repurposed in the clinic as anticancer agents, particularly in the case of tumours dependent on aberrant Notch signalling or with Notch-activating mutations (discussed in Section 2). Proteolytic cleavage of Notch receptors by the gamma-secretase complex is a prerequisite for the canonical activation of Notch transcriptional potential and subsequent signalling (unless downstream activating mutations are present), thus small-molecule GSIs have the potential to block this process. A major negative impact of targeting the gamma secretase complex with GSIs is that these therapies can lead to undesired on-target effects on tissues endogenously regulated by Notch, particularly the gut. Here, modulation of Notch 1 and 2 processing causes secretory goblet cell metaplasia. This leads to reduced differentiation and decreased numbers of the more absorptive cell lineages that should be present in the mucosa, resulting in intestinal toxicity [284]. Despite these limitations and adverse effects, pan-Notch GSIs have shown some clinical efficacy, with over 40 clinical trials, at different stages of completion investigating the use of GSIs in the treatment of cancer (Table 1).

#### 5.1.1. RO4929097

RO4929097 is a potent and selective small-molecule GSI that is orally bioavailable. It was originally developed by Roche for the treatment for Alzheimer’s disease; however, on-target effects on the Notch-signalling pathway led to its repositioning as a novel anticancer therapy, with preclinical studies demonstrating its antitumour efficacy in a range of cancer types, including lung and breast cancer, melanoma and glioblastoma [285,286,287,288]. A multitude of Phase I clinical studies indicated that RO4929097 has good tolerability as a single therapeutic agent and that it could also be safely used in combination with chemotherapy agents, radiotherapy and other molecular-targeted therapies [165,166,167,168,172,289,290] (Table 1). Subsequent reports from Phase II clinical trials of RO4929097 in advanced pancreatic adenocarcinoma (18 patients), melanoma (32 patients), metastatic colorectal cancer (37 patients) and platinum-resistant ovarian cancer (40 patients), indicate insufficient activity as single agents at the tested dosage regimens and a failure to reach study endpoints of clinical response [162,163,169,173]. A total of 12 Phase I and eight Phase II studies investigating the treatment of solid malignancies with RO4929097 as a monotherapy or in combination with standard chemotherapy or other targeted drugs (e.g., bevacizumab, erlotinib and vismodegib), have been withdrawn or stopped prematurely due to the decision by Roche to cease drug production and further clinical development following the poor results of Phase II trials. The last clinical trial with published results involving RO4929097 was a Phase I dose-escalation study to evaluate the safety and tolerability of combined RO4929097 and bevacizumab in the treatment of recurrent glioblastoma [170]. As discussed previously, Notch promotes tumour invasion and tumour-associated angiogenesis, thus there is a rationale for combining Notch inhibitors with antiangiogenic therapies to enhance their efficacy. While the combination of RO4929097 and bevacizumab was well-tolerated, a definitive maximum tolerated dose was not reached before Roche ceased production of the drug and terminated further clinical trials. Notably, none of the trials with RO4929097 involved genotype-selected cohorts with known Notch mutation status, thus future exploration of this GSI in Notch-activated tumours may prove more efficacious.

#### 5.1.2. LY3039478 (JSMD194)

LY3039478 is a potent GSI that has been shown preclinically to effectively inhibit Notch activity in multiple cell line models [223,224]. Initial results from a Phase I open-label, non-randomised, dose-escalation study of LY3039478 in the treatment of 110 advanced cancer patients, demonstrated that the drug was well-tolerated at doses associated with target engagement, as determined by Notch pathway gene expression analysis [175]. The Phase I Part B was used to confirm the recommended Phase II dose (RP2D) and included a number of soft tissue sarcoma (STS) and gastrointestinal stromal tumour (GIST) cases, which indicated the selected dose had a manageable safety profile and modest anticancer activity with a stabilised disease in 21–36% of cases [291]. Additionally, antitumour activity was clinically observed in breast cancer, leiomyosarcoma, and adenoid cystic carcinoma (ACC) [175]. Given the newly emerging role of Notch 1 as an oncogenic driver in ACC (discussed previously in Section 2.3.3), an expansion cohort of the Phase I trial was rolled out to confirm the RP2D effectiveness in 22 ACC patients and to document any observed antitumour activity [292]. As in the initial Phase I study, the safety profile of LY3039478 monotherapy was satisfactory and anti-Notch pharmacological activity was observed. While the heavily pretreated ACC patients did not indicate objective partial or complete responses, 58% showed disease stabilisation [292]. Further clinical development strategies should consider the treatment of ACC patients with known Notch-activating mutations. As described previously in regard to Notch’s involvement in glucocorticoid resistance, a Phase Ib study with the primary aim to evaluate the safety of LY3039478 in combination with dexamethasone in patients with T-ALL or T-LBL, indicated a good safety profile of this drug combination but no objective responses (NCT02518113). Another Phase Ib trial is exploring LY3039478 in combination with other anticancer agents (taladegib or abemaciclib or cisplatin/gemcitabine, or gemcitabine/carboplatin) in solid tumours including breast cancer, colon cancer, cholangiocarcinoma, and soft tissue sarcoma, is now completed with results yet to be reported (NCT02784795). While originally developed by Eli Lilly, LY3039478 was recently licensed to Juno Therapeutics under the new name JSMD194 to advance its treatment development programme of multiple myeloma. A number of publications have indicated that gamma secretase inhibition can boost the expression of a cancer marker called BCMA on the surface of cancer cells, particularly in multiple myeloma and this increases cancer cell susceptibility to BCMA-specific chimeric antigen receptor (CAR) T cell immunotherapy and subsequent tumour suppression [293]. Thus, Juno Therapeutics have initiated a Phase I of LY3039478/JSMD194 in combination with CAR T cell immunotherapy to treat relapsed or persistent multiple myeloma, estimated for primary completion in late 2021 (NCT03502577).

#### 5.1.3. PF-03084014 (Nirogacestat)

Nirogacestat or PF-03084014 is a potent, small-molecule, selective, noncompetitive GSI with antitumour and antimetastatic activity in numerous cancers including HCC [294]. It was granted Orphan Drug Designation (June 2018), and subsequently Fast Track Designation (November 2018) for the treatment of desmoid tumours, and Breakthrough Designation in mid-2019 for the treatment of adult patients with progressive, unresectable, recurrent or refractory to treatment desmoid tumours or deep fibromatosis [295]. This decision was made on the strength of promising findings in a Phase I trial of advanced solid tumours, including a cohort of desmoid tumours and a Phase II trial of 17 desmoid tumour patients, where the drug was overall well-tolerated and led to either partial response or disease stabilisation in all evaluable cases [177,179]. While the molecular mechanisms underlying the pathogenesis of these tumours is not fully understood, mutations in *APC* and *CTNNB1* leading to over-activation of the WNT/β-catenin pathway is common [296]. Critically, while the role of Notch in desmoid tumour progression is not well-understood, the WNT/β-catenin pathway exhibits crosstalk with Notch signalling [232] as described previously, demonstrating how nirogacestat may have clinical efficacy in this cancer type. It must also be noted that nirogacestat may have its therapeutic effect by inhibiting the cleavage of another substrate of gamma secretase, other than Notch. Nirogacestat is a drug previously developed by Pfizer that was licensed in 2017 to Pfizer spin-out company SpringWorks to advance the drug to the market for the treatment of this rare soft tissue cancer. A double-blind randomised Phase III trial, called the “DeFI trial”, evaluating nirogacestat in the treatment of desmoid tumour/aggressive fibromatosis, is currently recruiting patients (NCT03785964). An additional Phase II trial investigating this drug in the treatment of advanced and unresectable desmoid tumours in children and adolescents is due to begin recruitment in the second quarter of 2020 (NCT04195399). The outcome and objective response rates of these trials may lead to the first FDA approval of a Notch-targeted therapy and the first-ever FDA-approved treatment for desmoid tumours.

#### 5.1.4. AL101 (BMS-906024)

A small-molecule pan-Notch inhibitor of gamma secretase enzyme, BMS-906024, with favourable in vitro pharmacology and pharmacokinetics was developed by Bristol–Myers–Squibb and preclinical evaluations reported good antitumour efficacy in xenograft models of solid and haematological malignancies [297]. Three Phase I dose-escalation clinical trials investigating BMS-906024 alone or in combination with standard chemotherapy in the treatment of refractory haematological malignancies (T-ALL or T-LBL) or advanced solid tumours have been completed (NCT01292655, NCT01363817 and NCT01653470). Trial reports indicated that BMS-906024 had a good safety profile and promising antileukemic efficacy, with 8/25 patients showing at least a 50% reduction in bone-marrow-derived blasts [181]. Another follow-up drug from the same class of pan-Notch GSIs, called BMS-986115 was also identified and was investigated for safety and tolerability in the treatment of solid tumours in a Phase I clinical trial [183]. This trial was terminated early due to the relicensing of BMS-906024 and BMS-986115 to another drug company, Ayala Pharmaceuticals, in late 2017 for further clinical development under the new brand names AL101 and AL102, respectively [298]. A Phase I trial of BMS-906024/AL101 (NCT01292655) in patients with advanced or metastatic solid tumours, ACC, TNBC and NSCLC, was carried out to evaluate the safety and tolerability of two regimens (weekly or fortnightly intravenous dosing), as well as secondary outcomes of compound pharmacodynamics and pharmacokinetics. AL101 at the recommended Phase II dose was well-tolerated with expected gastrointestinal toxicity events, e.g., diarrhoea, being less frequent than those reported for other GSIs in clinical trials. Weekly dosing of AL101 led to continuous Notch inhibition (as measured by Notch-regulated *HES1* mRNA) and clinical activity against several tumour types. Of particular note, one ACC patient harbouring a *NOTCH1* gain-of-function or activating mutation had a complete partial response [180]. Preclinical data have shown that AL101 has significant antitumour efficacy in ACC PDXs harbouring Notch-activating mutations but not in ACC PDX models lacking mutations, providing proof-of-concept for targeting this ACC patient genotype [299]. Recruitment for the Phase II trial of AL101 in patients with unresectable ACC-bearing activating Notch mutations began in 2018 (ACCURACY trial, NCT03691207). The primary endpoint is objective response rate and secondary endpoints include adverse effects, OS and PFS. In 2019, AL101 was granted Orphan Drug Designation by the FDA for the treatment of Notch mutated ACC [300], and initial positive results from the ongoing Phase II trial [182] has led to its additional Fast Track Designation by the FDA in 2020 [301]. These designations will facilitate the progress of AL101 through the drug development pipeline and review process, making it available quicker to treat ACC patients, for which there are currently no FDA-approved treatments options. Currently, the clinical potential of AL101 is being explored in other cancer types with emerging preclinical data suggesting the AL101 can significantly impact tumour growth in TNBC PDX models harbouring Notch mutations and/or gene fusions, but not wild-type Notch [302]. This data will support the development of AL101 for the therapeutic indication of TNBC-bearing Notch-activating mutations.

### 5.2. DLL-4 Ligand-Targeted Antibodies

GSIs are characterised by their inherent ability to unselectively target Notch signalling regardless of the ligand and/or Notch receptor involved, meaning they can be effective in a wide spectrum of Notch-activated cancer. On the downside, their recognition of other noncancer context-specific substrates means they are associated with severe intestinal toxicity that needs to be managed carefully in clinical trials. A more targeted approach involves the use of monoclonal antibodies to block the Notch ligand–receptor interaction.

DLL-4 is critically important in vascular development, and in the context of cancer, its upregulation has been demonstrated in both tumour cells and associated tumour blood vessels. Inhibition of DLL-4 promotes nonproductive angiogenesis and tumour necrosis, effectively inhibiting tumour growth, which was demonstrated in several in vivo models of cancer. Several anti-DLL-4 monoclonal antibodies (demcizumab, enoticumab and MEDI0639) have been developed and progressed to various stages of clinical trials in advanced solid cancers including pancreatic, lung and ovarian cancer (see Table 1). Demcizumab, in combination with chemotherapy in Phase I clinical trials, was well-tolerated and had a satisfactory safety profile. A Phase Ib trial led by OncoMed evaluated the combination of demcizumab with a PD-1 receptor antagonist, pembrolizumab, in NSCLC and the combination was well-tolerated with mild adverse effects. Out of the 27 patients enrolled, one had a partial response and eight had stabilised disease; however, a lack of enhanced antitumour activity was reported [191]. A Phase II trial, named “Yosmite” (NCT02289898) investigating demcizumab in combination with chemotherapy, abraxane and gemcitabine, in pancreatic cancer failed to reach the primary outcome of PFS, with similar median PFS time recorded across all arms of the study [190,303]. A Phase Ib/II trial of demcizumab plus chemotherapy, carboplatin and pemetrexed, in the treatment of NSCLC also failed to meet the primary outcome of an overall response rate (ORR; 28% in the demcizumab plus chemotherapy group versus 52% in the chemotherapy only group) [189]. Based on the lack of benefit over standard-of-care, OncoMed halted all further clinical development of demcizumab. No progress with other DLL-4 antibodies has been reported elsewhere.

### 5.3. Notch-Receptor-Targeted Antibodies

A second strategy to block Notch signalling is the development of monoclonal antibodies directed specifically at the Notch receptor. Brontictuzumab (OMP-52M51) is a humanised IgG2 antibody generated by immunising mice with a fragment of the LNR and NRR domains of the Notch 1 receptor. It has shown efficacy in T-ALL and MCL in vitro and in vivo models, and in leukaemia PDX models harbouring two of the common Notch 1 activating mutations, i.e., mutations in the HD and PEST domains [304,305]. A Phase I dose-escalation clinical trial by OncoMed of brontictuzumab in patients with haematological malignancies and known Notch 1 mutation status was completed in 2016, reporting that the therapy was generally well-tolerated but had limited antitumour activity [192]. In this trial, three out of the 24 patients had a Notch 1 activating mutation and one of these patients was reported to have stabilised disease. A Phase I dose-escalation study of the same drug was also completed in solid tumours and demonstrated similar well-tolerated safety profile (diarrhoea was the main dose-limiting toxicity (DLT) reported). Brontictuzumab was noted to have antitumour efficacy in a subcohort of patients with Notch-activated ACC [193,194]. Further studies targeting Notch 1 in a genotype-defined ACC patient population should be a logical next step in the clinical development of brontictuzumab; however, there are no reports currently of such plans. Disappointingly, a Phase Ib trial of brontictuzumab in combination with chemotherapy as a third-line treatment for advanced colorectal cancer (NCT03031691) was terminated after five months, with a final enrolment of only seven patients, due to intolerable toxicities associated with the drug combination [195].

Another antibody, called OMP-59R5 (tarextumab) has been developed by OncoMed that selectively targets Notch 2 and Notch 3. In preclinical studies, this therapy has shown notable antitumour efficacy in combination with gemcitabine and nab-paclitaxel, in PDX models of several solid tumour types including breast, pancreatic, lung and ovarian cancer [306]. Interestingly, tarextumab was found to inhibit Notch 2/3 activity and Notch target gene expression in both human-derived cancer cells and the mouse-derived stromal cells in the xenografts, suggesting an interaction with multiple cell types. It demonstrated antiproliferative and anti-CSC activity in xenografts. In a Phase I dose-escalation study in the treatment of advanced solid tumours, tarextumab was reported to be well-tolerated overall (the most common adverse symptom was gastrointestinal toxicity-associated diarrhoea in 83% of patients) and biomarker analysis indicated the clinical doses were adequate to inhibit Notch gene signalling [196]. OncoMed have since led two Phase II clinical trials, ALPINE and PINNCLE, investigating the use of tarextumab in combination with standard chemotherapy agents in the treatment of pancreatic cancer and NSCLC, respectively. In the Phase Ib ALPINE trial, which enrolled 35 patients, tarextumab was generally well-tolerated in patients and demonstrated an encouraging correlation between antitumour activity and Notch 3 activity [197]. The Phase II portion of this trial, which recruited 177 patients, failed to reach the study endpoint of OS or the secondary endpoints of PFS, ORR and Notch biomarker activity; in fact, tarextumab treated patients had significantly worse outcomes than those on the placebo treatment arm [198]. Encouraging reports of the Phase Ib PINNACLE trial indicated a dose-efficacy association with a survival benefit in a subgroup of patients (15 of 27 patients enroled) treated with a higher dose of tarextumab, in addition to a manageable safety profile [199]. However, the randomised Phase II trial with 145 patients, failed to reach the primary endpoint of PFS, with tarextumab showing no benefit over placebo treatment in this larger study, and biomarker analysis failed to define a Notch-activated patient subset where treatment improved median OS or PFS (NCT01859741) [195]. Overall, the future of brontictuzumab and tarextumab’s clinical use in combination with the standard-of-care in difficult-to-treat cancers is not promising, given the lack of supporting clinical data currently.

### 5.4. Inhibition of the Notch Transcription Complex

There have been efforts to target the further downstream signalling activities of intracellular Notch rather than preventing the activation of Notch itself, potentially avoiding some of the associated toxicities. Uniquely, Notch signalling can be blocked regardless of any genetic activating mutations in the receptor. As described previously, once cleavage of Notch 1 occurs, NICD is released and translocates to the nucleus where it forms a transcription complex with RBP-J/CSL DNA-binding protein and subsequently mastermind-like (MAML) adaptor protein (Figure 1).

A synthetic, cell-permeable, α-helical peptide (SAHM1) has been developed that blocks MAML1 recruitment with high affinity for the interface on the Notch-CSL/RBP-J transactivation complex effectively reducing T-ALL cell line proliferation and Notch-driven progression of leukaemia in a mouse model of T-ALL [307]. While this approach has the potential to be a more specific Notch targeting approach, with efficacy in pathologies such as asthma and eye disorders [308,309], there is currently a lack of pharmacokinetic and pharmacodynamic data to support a role in the cancer setting. Aside from this peptide-based therapeutic approach, a first-in-class small-molecule inhibitor of Mastermind recruitment-1 (IMR-1) has been identified from in-silico screening of over 1.5 million drug-like compounds. It blocks recruitment of MAML-1 to the Notch transcription activation complex (NTC) on chromatin in vitro with a dose-dependent decrease in Notch target gene transcription (e.g., *HES1* and *HEYL*) similar to the GSI, DAPT [310]. IMR-1 demonstrated efficacy in xenograft tumour models, without any adverse effects on animal weight or other vital parameters [310]. Despite these promising preclinical findings, a lack of further pharmacokinetic and pharmacodynamic studies has impeded the continued progress of IMR-1 as a novel therapy for cancer.

Recently a first-in-class novel small-molecule inhibitor of RBP-J, RIN1, has been identified by high throughput screening that can block the interaction of NICD with RBP-J [311]. It also blocks the interaction between RBP-J and a nonrelated scaffold protein called SHARP, involved in its transcription suppressor role in the absence of Notch signalling. RIN1 treatment resulted in gene expression changes more akin to RBP-J siRNA knockdown rather than bona fide Notch inhibition typically observed following treatment with the GSI, DAPT. Thus, the clinical utility of RIN1 is not clear given the inhibition of RBP-J in both its transcription repressing and activating contexts. However, it may be a useful exploratory tool for understanding further the interaction of Notch and RBP-J.

CB-103 (Cellestia Biotech AG) is a first-in-class orally available small molecule PPI (protein-protein interactor) pan-Notch inhibitor that interrupts the assembly of the Notch transcription complex on DNA within the nucleus, leading to downregulation of Notch transcriptional effectors including *MYC*, *CCND1* and *HES1*. In vitro pharmacodynamics testing of CB-103 indicated an inhibition of Notch signalling in a dose-dependent manner. In a panel of over 120 cell lines, CB-103 showed efficacy in a subset of 24 cell lines spread across numerous haematological (lymphomas and leukaemias) and solid malignancies (lung, breast and sarcoma), in in vivo and PDX models, in addition to an excellent safety profile [312,313]. Furthermore, the aggressive TNBC cell line HCC1187, which harbours a chromosomal translocation in the Notch 2 gene, rendering it resistant to GSIs, demonstrated sensitivity to CB-103, suggesting that it may be a more suitable treatment strategy for Notch-addicted tumours with such genotypes. A first-in-man Phase I/IIa multicentre open-label dose-escalation trial with an expansion study to determine preliminary antitumour efficacy is ongoing currently (NCT034226790), aiming to recruit 165 patients with advanced, refractory or metastatic solid tumours (breast, colorectal, cholangiocarcinoma and sarcoma) or haematological malignancies for whom no standard therapy exists [200].

To date, in comparison to the preclinical and clinical development of GSIs, the development of alternative therapeutic peptides or small-molecule inhibitors that directly target the intracellular Notch pathway or NTC has been slow to gather momentum. The outcome of the Phase II CB-103 clinical trial, estimated for completion in mid-2021, should provide critical data on safety profile and efficacy with potential clinical utility and may encourage increased interest in this under-researched field.

## 6. Conclusions

The role of Notch as an oncogene in the context of certain cancer types has been well-established over the last 20 years. A wide range of mutations and gene alterations have been reported in the literature that results in Notch receptor overactivation. Hotspot mutations in the HD and PEST domains of Notch receptors originally identified in T-ALL are now also prominent in several solid malignancies including breast, lung, colorectal cancer and adenoid cystic carcinoma. Overexpression of Notch signalling in the absence of known gene alterations or mutations is also evident in a number of cancer types including melanoma, ovarian cancer, pancreatic cancer and prostate cancer, indicating the large therapeutic scope of Notch targeting therapies. Many studies support a critical role of Notch in response to radiation and chemotherapy (particularly with regards to CSC renewal), hormone therapy and other molecular-targeted therapies such as anti-EGFR and anti-PI3K molecular-targeted therapies. Furthermore, treatment-resistant cancer can often be resensitised to standard treatments by combining the therapy in question with a Notch pathway inhibitor, suggesting a critical role of Notch in acquired treatment resistance mechanisms. Thus, combination treatments with Notch inhibitors are a promising therapeutic strategy, which should be further investigated in the clinical setting.

As described previously, efforts to antagonise Notch have involved blocking ligand binding (e.g., DLL-4 antagonists; demcizumab and enoticumab), blocking Notch receptor activation (e.g., brontictuzumab and tarextumab), blocking the generation of NICD by the gamma secretase complex (e.g., GSIs; RO4929097, LY3039478/JMSD194, nirogacestat and AL101) and most recently by blocking the Notch transcription complex from binding to DNA (e.g., CB-103). However, a number of trials have reported a lack of efficacy and adverse effects with Notch targeting in cancer.

In order to achieve satisfactory clinical efficacy with Notch inhibition, careful consideration must be given to who is selected for treatment and when the treatment should be started. There are currently no established biomarkers to predict response to anti-Notch therapies. In fact, the majority of ligand/receptor antibodies or GSIs in Phase I and II trials have recruited nonstratified populations where the Notch mutation and/or activation status is not known. This may be a reason for failure to reach endpoints of PFS and ORR. Furthermore, Notch activation may not become evident until resistance to primary treatment occurs, if Notch is playing a role in driving resistance mechanisms in a particular tumour type. Thus, it will be important to monitor Notch levels in such patients, to determine whether they would benefit from combined or second-line treatment with a Notch antagonist. GSIs, which nonselectively target gamma secretase complexes with many substrates beyond the Notch receptor family, are associated with dose-limiting toxicities that need to be managed carefully. While Notch ligand/receptor antibodies, should offer a more specific targeted approach, they still have associated dose-limiting toxicities (likely due to on-target interaction with Notch signalling in noncancerous tissues). Thus, carefully planned, short, intermittent dosing regimens may sustain efficacy and help to mitigate toxicity.

The recent FDA Orphan Drug Designation and Fast Track Designation of the GSIs, nirogacestat and AL101, for the treatment of desmoid tumours and Notch-mutant ACC respectively have made promising and positive progress in this field of research. Interestingly, other clinical trials of the anti-Notch 1 receptor antagonist such as brontictuzumab and the GSI LY3039478 also demonstrated antitumour activity or disease stabilisation in small numbers of ACC patients, further supporting the rationale for Notch-mutant ACC patients as likely candidates responsive to Notch therapy. Notably, most clinical trials with Notch antagonists to date have enrolled recurrent, heavily pretreated chemo-resistant cancer patients, which are difficult to treat in clinical trials. Thus, further clinical development strategies for Notch antagonists should consider the first-line treatment of ACC patients harbouring known Notch-activating mutations in combination with standard therapy, focusing on the endpoints of confirmed Notch target engagement and antitumour responses e.g., PFS and ORR.

## Figures and Tables

**Table 1:** A current summary of cancer clinical trials involving Notch antagonists.

Drug Class	Drug Name	CT Identifier	Study Name	Phase	Primary Endpoint	Status	Clinical Outputs Met?	Published Results
**Gamma Secretase Inhibitor** **(GSI)**	**RO4929097** **(R4733)**	NCT00532090	A Multiple Ascending Dose Study of R4733 in Patients with Advanced Solid Tumours	I	AE	Completed	Yes	No results published
		NCT01070927	An Exploratory Study of RO4929097 in Patients with Recurrent or Refractory Non-Small Cell Lung Cancer	II	PD	Completed	Yes	No results published
		NCT01071564	RO4929097 and Vismodegib in Treating Patients with Breast Cancer That is Metastatic or Cannot Be Removed By Surgery	I	MTD, AE, DLT	Terminated early (drug development and manufacturing ceased)		No results published
		NCT01088763	Gamma-Secretase Inhibitor RO4929097 in Treating Young Patients with Relapsed or Refractory Solid Tumours, CNS Tumours, Lymphoma, or T-Cell Leukemia	I	MTD, DLT	Terminated		No results published
		NCT01096355	Gamma-Secretase Inhibitor RO4929097 in Treating Patients with Metastatic or Unresectable Solid Malignancies	I	DLT	Completed	Yes	No results published
		NCT01116687	RO4929097 in Treating Patients with Metastatic Colorectal Cancer	II	ORR	Completed	No	[162]
		NCT01119599	RO4929097, Temozolomide, and Radiation Therapy in Treating Patients with Newly Diagnosed Malignant Glioma	I	MTD	Completed	Yes	www.clinicaltrials.gov
		NCT01120275	Gamma-Secretase/Notch Signalling Pathway Inhibitor RO4929097 in Treating Patients with Stage IV Melanoma	II	PFS, OS	Terminated early (drug development and manufacturing ceased)		[163]
		NCT01122901	Gamma-Secretase/Notch Signalling Pathway Inhibitor RO4929097 in Treating Patients with Recurrent or Progressive Glioblastoma	II	PFS, PD	Terminated early (drug development and manufacturing ceased)		[164]
		NCT01131234	Gamma-Secretase Inhibitor RO4929097 and Cediranib Maleate in Treating Patients with Advanced Solid Tumours	I	MTD	Completed	Yes	[165]
		NCT01141569	A Study of RO4929097 in Patients with Advanced Renal Cell Carcinoma That Have Failed Vascular Endothelial Growth Factor (VEGF)/Vascular Endothelial Growth Factor Receptor (VEGFR) Therapy	II	ORR	Completed	No	www.clinicaltrials.gov
		NCT01145456	Gamma-Secretase Inhibitor RO4929097 and Gemcitabine Hydrochloride in Treating Patients with Advanced Solid Tumours	I	DLT	Completed	Yes	[166]
		NCT01149356	RO4929097 And Exemestane in Treating Pre- and Postmenopausal Patients with Advanced or Metastatic Breast Cancer	I	AE, Time to relapse	Terminated early (drug development and manufacturing ceased)		[167]
		NCT01151449	Gamma-secretase/Notch Signalling Pathway Inhibitor RO4929097 in Treating Patients with Advanced, Metastatic, or Recurrent Triple Negative Invasive Breast Cancer	II	ORR, PFS	Terminated (Administratively Completed)		www.clinicaltrials.gov
		NCT01154452	Vismodegib and Gamma-Secretase/Notch Signalling Pathway Inhibitor RO4929097 in Treating Patients with Advanced or Metastatic Sarcoma	I/II	I: MTD II: PFS	Completed	I: Yes II: No	I: [153] II: www.clinicaltrials.gov
		NCT01158274	RO4929097 and Capecitabine in Treating Patients with Refractory Solid Tumours	I	MTD, AE	Completed	Yes	[168]
		NCT01175343	A Phase II Study of RO4929097 in Advanced Platinum Resistant Ovarian Cancer	II	PFS, PD -CA125 progression	Completed	No	[169]
		NCT01189240	RO4929097and Bevacizumab in Treating Patients with Progressive or Recurrent Malignant Glioma	I/II	MTD	Terminated early (drug development and manufacturing ceased)		[170]
		NCT01193868	RO4929097 in Treating Patients with Advanced Non-Small Cell Lung Cancer Who Have Recently Completed Treatment With Front-Line Chemotherapy	II	ORR	Completed	N/A	No results published
		NCT01208441	RO4929097 and Letrozole in Treating Post-Menopausal Women with Hormone Receptor-Positive Stage II or Stage III Breast Cancer	Ib	MTD	Terminated early (drug development and manufacturing ceased)		No results published
		NCT01200810	Bicalutamide and RO4929097 in Treating Patients with Previously Treated Prostate Cancer	II	PD - PSA Progression	Terminated early (drug development and manufacturing ceased)		[171]
		NCT01198535	Phase I Study of Cetuximab With RO4929097 in Metastatic Colorectal Cancer	I	MTD	Terminated early		No results published
		NCT01198184	Gamma-Secretase/Notch Signalling Pathway Inhibitor RO4929097 and Temsirolimus in Treating Patients with Advanced Solid Tumours	I	DLT, AE	Completed	Yes	[172]
		NCT01196416	Gamma-secretase/Notch Signalling Pathway Inhibitor RO4929097 in Combination with Cisplatin, Vinblastine, and Temozolomide in Treating Patients With Recurrent or Metastatic Melanoma	I/II	ORR, MTD, OS	Completed	No	[163]
		NCT01193881	RO4929097 and Erlotinib Hydrochloride in Treating Patients with Stage IV or Recurrent Non-small Cell Lung Cancer	I	AE, MTD, PD	Terminated early (drug development and manufacturing ceased)		No results published
		NCT01192763	RO4929097 Before Surgery in Treating Patients with Pancreatic Cancer NCT01192763	I	PD, AE	Terminated early (drug development and manufacturing ceased)		www.clinicaltrials.gov
		NCT01217411	RO4929097 and Whole-Brain Radiation Therapy or Stereotactic Radiosurgery in Treating Patients with Brain Metastases From Breast Cancer	I	MTD, ORR	Terminated (slow patient enrolloment and drug development ceased)		N/A—study too small
		NCT01216787	RO4929097 in Treating Patients with Stage IIIB, Stage IIIC, or Stage IV Melanoma That Can Be Removed by Surgery	II	PD	Withdrawn (drug development and manufacturing ceased)		N/A
		NCT01218620	Gamma-Secretase/Notch Signalling Pathway Inhibitor RO4929097 in Treating Patients with Advanced Solid Tumours	I	PD	Completed	N/A	No results published
		NCT01232829	Gamma Secretase Inhibitor RO4929097 in Previously Treated Metastatic Pancreas Cancer	II	OS	Completed	No	[173]
		NCT01236586	RO4929097 in Children with Relapsed/Refractory Solid or CNS Tumours, Lymphoma, or T-Cell Leukemia	I/II	I: MTD, AE II: Efficacy	Withdrawn (drug development and manufacturing ceased)		N/A
		NCT01238133	Gamma-Secretase/Notch Signalling Pathway Inhibitor RO4929097, Paclitaxel, and Carboplatin Before Surgery in Treating Patients with Stage II or Stage III Triple-Negative Breast Cancer	I	DLT, MTD	Terminated early (drug development and manufacturing ceased)		[174]
		NCT01251172	RO4929097 After Autologous Stem Cell Transplant in Treating Patients with Multiple Myeloma	II	ORR	Withdrawn (drug development ceased)		N/A
		NCT01270438	Combination Chemotherapy and Bevacizumab with or Without RO4929097 in Treating Patients With Metastatic Colorectal Cancer	II	PFS	Withdrawn (drug development ceased)		N/A
		NCT01189240	RO4929097 and Bevacizumab in Treating Patients with Progressive or Recurrent Malignant Glioma	I	DLT, MTD	Terminated early (drug development ceased)		[170]
	**LY3039478** **(JSMD194)**	NCT01695005	A Study of LY3039478 in Participants with Advanced Cancer	I	DLT, ORR	Completed	Yes	[175]
		NCT02518113	A Study of LY3039478 in Combination with Dexamethasone in Participants With T-ALL/T-LBL	I	DLT, AE, ORR	Completed	No	www.clinicaltrials.gov
		NCT02784795	A Phase 1b Study of LY3039478 in Combination with Other Anticancer Agents in Patients With Advanced or Metastatic Solid Tumours	I	MTD	Completed	Yes	No results published
		NCT03502577	BCMA-Specific CAR T-Cells Combined with a Gamma Secretase Inhibitor (JSMD194) to Treat Relapsed or Persistent Multiple Myeloma	I	MTD, AE	Ongoing		N/A
	**Nirogacestat** **(PF-03084014)**	NCT00878189	A Phase I trial of PF-03084014 in patients with advanced solid tumour malignancy and T-cell acute lymphoblastic leukemia/lymphoblastic lymphoma	I	DLT	Completed	Yes	[176] [177]
		NCT01876251	A Study Evaluating The PF-03084014 In Combination with Docetaxel In Patients With Advanced Breast Cancer	I	DLT, PFS	Discontinued (change in development strategy)		[178]
		NCT02299635	Study of PF-03084014 In Combination with Gemcitabine And Nab-Paclitaxel In Patients With Metastatic Pancreatic Adenocarcinoma Not Previously Treated With Anticancer Therapies	II	ORR	Discontinued (change in development strategy)		Data on primary endpoint not collected
		NCT02109445	Study of PF-03084014 In Combination with Gemcitabine And Nab-Paclitaxel In Patients With Metastatic Pancreatic Adenocarcinoma Not Previously Treated With Anticancer Therapies	I/II	I: DLT II: OS	Discontinued (change in development strategy)		Data on primary endpoint not collected. Phase II not initiated
		NCT01981551	Phase II Trial of the Gamma-Secretase Inhibitor PF-03084014 in Adults with Desmoid Tumours/Aggressive Fibromatosis	II	ORR	Active		[179]
		NCT03785964	A Randomized, Double-Blind, Placebo-Controlled, Phase 3 Trial of Nirogacestat Versus Placebo in Adult Patients with Progressing Desmoid Tumours/Aggressive Fibromatosis (DeFi)	III	PFS	Recruiting		N/A
		NCT04195399	A Safety, Pharmacokinetic and Efficacy Study of a y-Secretase Inhibitor, Nirogacestat (PF-03084014), in Children and Adolescents with Progressive, Surgically Unresectable Desmoid Tumours	II	PFS, DLT, PD	Recruiting 2020		N/A
	**AL101** **(BMS-906024)**	NCT01292655	Study to Evaluate the Safety and Tolerability of IV Doses of BMS-906024 in Subjects with Advanced or Metastatic Solid Tumours	I	AE, DLT	Completed	Yes	[180]
		NCT01363817	Study to Evaluate the Safety and Tolerability of Weekly Intravenous (IV) Doses of BMS-906024 in Subjects with Acute T-cell Lymphoblastic Leukemia or T-cell Lymphoblastic Lymphoma	I	AE, DLT	Completed	Yes	[181]
		NCT01653470	Study to Evaluate Safety & Tolerability of BMS-906024 in Combination With Chemotherapy & to Define DLTs & MTD of BMS-906024 in Combination With One of the Following Chemotherapy Regimens; Weekly Paclitaxel, 5FU+Irinotecan or Carboplatin+Paclitaxel in Subjects With Advanced / Metastatic Solid Tumours	Ib	AE, DLT	Completed	Yes	No results published
		NCT03691207	A Study Of AL101 In Patients With Adenoid Cystic Carcinoma (ACC) Bearing Activating Notch Mutations (ACCURACY)	II	ORR	Ongoing		[182]
	**AL102** **(BMS-986115)**	NCT01986218	Phase I Ascending Multiple-Dose Study of BMS-986115 in Subjects with Advanced Solid Tumours	I	DLT, AE	Completed	Yes	[183]
**DLL-4 Antibody**	**MEDI0639**	NCT01577745	A Phase 1 Study to Evaluate the Safety, Tolerability, and Pharmacokinetics of MEDI0639 in Advanced Solid Tumours	I	DLT, MTD	Completed	Yes	[184]
	**Enoticumab (REGN421)**	NCT00871559	A Multiple-Ascending-Dose Study of the Safety and Tolerability of REGN421 (SAR153192) in Patients with Advanced Solid Malignancies	I	DLT	Completed	Yes	[185]
	**Demcizumab**	NCT01189929	A Study of Gemcitabine and Demcizumab (OMP-21M18) With or Without Abraxane^®^ as 1st-line Treatment in Subjects with Locally Advanced or Metastatic Pancreatic Cancer	Ib	MTD	Completed	Yes	[186]
		NCT01189968	A Study of Carboplatin and Pemetrexed Plus Demcizumab (OMP-21M18) in Subjects with Non-Squamous Non-Small Cell Lung Cancer	Ib	MTD	Completed	Yes	[187]
		NCT01952249	A Study of Demcizumab Plus Paclitaxel in Subjects with Platinum Resistant Ovarian (SIERRA)	Ib	DLT, MTD	Completed (Phase II portion planned but not initiated)	Yes	[188]
		NCT02259582	A Study of Carboplatin, Pemetrexed Plus Placebo vs Carboplatin, Pemetrexed Plus 1 or 2 Truncated Courses of Demcizumab in Subjects with Non-Squamous Non-Small Cell Lung Cancer (DENALI)	II	ORR	Completed	No	[189]
		NCT02289898	Study of Gemcitabine, Abraxane^®^ Plus Placebo Versus Gemcitabine, Abraxane^®^ Plus 1 or 2 Truncated Courses of Demcizumab in Subjects With 1st-Line Metastatic Pancreatic Ductal Adenocarcinoma (YOSEMITE)	II	PFS	Completed	No	[190]
		NCT02722954	A Phase 1b Study of Demcizumab Plus Pembrolizumab in Locally Advanced or Metastatic Solid Tumours	Ib	DLT	Completed	Yes	[191]
**Notch receptor Antibody**	**Brontictuzumab** **(OMP-52M51)**	NCT01703572	A Dose Escalation Study of OMP-52M51 in Subjects with Lymphoid Malignancies	I	DLT	Completed	Yes	[192]
		NCT01778439	A Dose Escalation Study of OMP-52M51 in Subjects with Solid Tumours	I	DLT	Completed	Yes	[193]
		NCT02662608	Compassionate Use of Brontictuzumab for Adenoid Cystic Carcinoma (ACC)	N/A	PFS	Completed	No	[194]
		NCT03031691	A Study of Brontictuzumab with Chemotherapy for Subjects with Previously Treated Metastatic Colorectal Cancer	Ib	AE, DLT, immuno-genicity	Terminated early		[Press release] [195]
	**Tarextumab (OMP-59R5)**	NCT01277146	A Dose Escalation Study of OMP-59R5 in Subjects with Solid Tumours	I	AE, DLT	Completed	Yes	[196]
		NCT01647828	A Phase 1b/2 Study of OMP-59R5 in Combination with Nab-Paclitaxel and Gemcitabine in Subjects With Previously Untreated Stage IV Pancreatic Cancer (ALPINE)	I/II	I: DLT, II: PFS	Completed	I: Yes II: No	I: [197] II: [198]
		NCT01859741	A Phase 1b/2 Study of OMP-59R5 (Tarextumab) in Combination with Etoposide and Platinum Therapy (PINNACLE)	I/II	I: MTD, ORR II: PFS	Terminated early		I: [199] II: [195]
**Notch Transcription** **Complex Inhibitor**	**CB-103**	NCT03422679	Study of CB-103 in Adult Patients with Advanced or Metastatic Solid Tumours and Haematological Malignancies	I/II	I: DLT, II: Efficacy	Ongoing		[200]
		NCT03422679	Study of CB-103 in Adult Patients with Advanced or Metastatic Solid Tumours and Haematological Malignancies	I/II	I: DLT, II: Efficacy	Ongoing		[200]

AE: Adverse Effects, DLT: Dose-Limiting Toxicities, MTD: Maximum Tolerated Dose, ORR: Objective/Overall Response Rate, OS: Overall Survival, PD: Drug Pharmacodynamics Measurements, PFS: Progression-Free Survival, N/A: Not Applicable; No Data published.
